# Walking the Plank: An Experimental Paradigm to Investigate Safety Voice

**DOI:** 10.3389/fpsyg.2019.00668

**Published:** 2019-04-02

**Authors:** Mark C. Noort, Tom W. Reader, Alex Gillespie

**Affiliations:** Department of Psychological and Behavioural Science, London School of Economics and Political Science, London, United Kingdom

**Keywords:** safety voice, safety silence, safety concerns, experimental methodologies, behavioural observations

## Abstract

The investigation of people raising or withholding safety concerns, termed safety voice, has relied on report-based methodologies, with few experiments. Generalisable findings have been limited because: the behavioural nature of safety voice is rarely operationalised; the reliance on memory and recall has well-established biases; and determining causality requires experimentation. Across three studies, we introduce, evaluate and make available the first experimental paradigm for studying safety voice: the “Walking the plank” paradigm. This paradigm presents participants with an apparent hazard (walking across a weak wooden plank) to elicit safety voice behaviours, and it addresses the methodological shortfalls of report-based methodologies. Study 1 (*n* = 129) demonstrated that the paradigm can elicit observable safety voice behaviours in a safe, controlled and randomised laboratory environment. Study 2 (*n* = 69) indicated it is possible to elicit safety silence for a single hazard when safety concerns are assessed and alternative ways to address the hazard are absent. Study 3 (*n* = 75) revealed that manipulating risk perceptions results in changes to safety voice behaviours. We propose a distinction between two independent dimensions (concerned-unconcerned and voice-silence) which yields a 2 × 2 safety voice typology. Demonstrating the need for experimental investigations of safety voice, the results found a consistent mismatch between self-reported and observed safety voice. The discussion examines insights on conceptualising and operationalising safety voice behaviours in relationship to safety concerns, and suggests new areas for research: replicating empirical studies, understanding the behavioural nature of safety voice, clarifying the personal relevance of physical harm, and integrating safety voice with other harm-prevention behaviours. Our article adds to the conceptual strength of the safety voice literature and provides a methodology and typology for experimentally examining people raising safety concerns.

## Introduction

The term safety voice describes the behaviour of raising, or withholding, safety concerns to prevent physical harm from hazardous situations (e.g., Tucker et al., 2008). Across organisational (e.g., healthcare, energy), family (e.g., transport, DIY), and leisure contexts (e.g., high risk sports), promoting the act of raising of safety concerns can reduce people's exposure to hazards (e.g., medicine dispensation, dangerous driving, high-altitude climbing without proper gear), and prevent physical harm (Anicich et al., 2015; Manias, 2015). The absence of speaking-up, also termed safety silence (Barzallo Salazar et al., 2014), has been implicated in catastrophes such as the 1983 Challenger disaster (Moorhead et al., 1991) and 2010 Deepwater horizon oil spill (Reader and O'Connor, 2014), and is estimated to be involved in 25% of aviation accidents (Tarnow, 1999; Bienefeld and Grote, 2012).

Due to the difficulty of observing safety voice in safety-critical situations, academic safety voice publications tend to present data obtained through report-based data (e.g., surveys, focus groups, interviews, vignettes; Noort et al., submitted) in which individuals or their seniors report on behavioural responses to previously held or imagined safety concerns. Yet, it remains unclear whether data from reports is reflective, explanatory, and predictive of safety voice behaviours. Alternative approaches are required to study the conditions and ways through which people raise or withhold safety concerns, and to address this, we propose and test the first experimental paradigm for investigating safety voice. Through investigating the occurrence of safety voice behaviours in a laboratory setting, and the challenges in assessing these, we aim to establish a methodology for (i) observing the behavioural nature of safety voice; (ii) reducing the methodological reliance on memory and imagination; and (iii) advancing knowledge on the factors that predict safety voice.

### Safety Voice: The Need for an Experimental Approach

The term “safety voice” is used as a broad label to, confusingly, encompass a behaviour and its counterpart: safety voice (i.e., raising safety concerns) and safety silence (Van Dyne et al., 2003; Okuyama et al., 2014; Manapragada and Bruk-lee, 2016; Morrow et al., 2016). “Safety voice” often relates to raising safety concerns, which is the act of speaking-up about safety issues, through informal or formal communication channels, to a variety of targets (e.g., management, co-workers, the public), with the intention to mitigate harm from a situation perceived to be dangerous (Tucker et al., 2008). Through doing this, people communicate safety issues with the aim of creating a shared perception of the risk and, ultimately, avoiding the danger (Okuyama et al., 2014). Safety silence, the “non-voicing” type of safety voice, is defined as the active withholding of safety concerns (e.g., Okuyama et al., 2014), and is thus different from the simple absence of speaking-up: this can follow from not having safety concerns (i.e., “unconcerned silence”).

The concept of safety voice emerged from the literature on employee voice and silence (Van Dyne et al., 2003; Morrison, 2011, 2014), and appears similar. Yet, voice behaviours (in the broadest sense) can be distinguished based on message content (e.g., Morrison, 2011; Liang et al., 2012), and the safety voice literature is characterised by a narrower concern (i.e., limited to prohibiting harm from safety issues), broader application (i.e., beyond organisational environments), more severe outcomes (e.g., fatalities), and has established different antecedents across levels of analysis (e.g., expected impact of harm, safety knowledge, workload, national culture; Noort et al., submitted). The message content of safety voice relates to the avoidance of harm based on perceived risks, and arguably types of harm may be distinguished: the prevention of physical (e.g., injuries, accidents), psychological (e.g., bullying, harassment), social (e.g., ostracism, unpleasant interactions) or ethical harm (e.g., loss of autonomy; Marshall, 1996). These issues are important to safety voice researchers and practitioners as they can contribute to unsafe outcomes (e.g., bullying can create a poor safety culture), yet physical harm may be easiest to operationalise (i.e., it is closer to a hazard, less ambiguous, easiest to manipulate), and other types of harm may occur beyond (potential) hazards.

Researching safety voice for academic or practice-based purposes is complex due to the elusive and sensitive nature of the phenomenon. Safety voice is a spontaneous response to hazards occurring in natural environments (e.g., wobbly stepladders, incorrect aircraft atmospheric pressure settings), and systematic behavioural observations can provide valuable insights into the dynamic social and physical context in which people raise safety concerns (Reiss, 1971; Mulhall, 2003; van Schagen and Sagberg, 2012; Rydenfält et al., 2015), real-time patterns of behaviour (e.g., attention; Waller and Kaplan, 2016; Lappi et al., 2017), demographic variations (Pérez-Tejera et al., 2018) or how people feel and act when they speak-up without having to rely on *post-hoc* reports (e.g., Mastrofski et al., 1998; Murphy and Dingwall, 2007), and may reveal stronger effects (Brodin et al., 2016). Yet, within natural environments, it is difficult to (i) observe short-lived and spontaneous behaviours that may not occur frequently (Mastrofski et al., 2010) in a resource efficient way (i.e., many resources are needed to capture brief moments of speaking-up/remaining silent; Reiss, 1971), (ii) record behaviours in a standardised way (e.g., across unsafe situations), (iii) assess the riskiness of a situation and whether people are withholding a safety concern (or did not understand the gravity of the situation), or (iv) ensure participants are not changing their natural behaviour (Nichols and Maner, 2008). A notable exception to these limitations of naturalistic observations are cockpit voice recordings, but to-date they have received limited empirical study in terms of safety voice (cf. U. Fischer and Orassanu, 2000).

To overcome the challenges of observing safety voice, practice-based investigations (e.g., inquiries, accident investigations; Rogers, 1986; Francis, 2013, 2015) and the vast majority of academic investigations into safety voice (i.e., a systematic review indicated 76% of academic publications; Noort et al., submitted) utilise methodologies that obtain data from participant reports on whether they or their supervisees raised or withheld safety concerns. For example, through participants providing statements during inquiries (e.g., Francis, 2015), stating their imagined response to a vignette scenario (Schwappach and Gehring, 2014c), recalling scenarios in which they held a safety concern and communicated this to others (e.g., Schwappach and Gehring, 2014d), or completing survey scales that elicit agreement with statements about imagined or generic scenarios (e.g., “I chose to remain silent when I have concerns about patient safety”; Delisle et al., 2016; Gkorezis et al., 2016). Applications of these methodologies for academic and non-academic purposes have enabled the identification of lay rationales for safety voice, contributing factors to major incidents, cross-sectional comparisons (e.g., across organisational departments), and testing of interventions to alter lay perceptions of the likelihood, with practitioners supplementing academic conclusions through providing better access to people involved in incidents, subject-matter experts, and faster publication of lessons learned (when conclusions are published).

However, there are limitations in the use of report-based methodologies to investigate safety voice. Reports have limited applicability for addressing situational factors (e.g., personal relevance of risk, group dynamics, previous history of raising safety concerns) and mechanisms (e.g., decision-making on risk) that can shape safety voice, and perhaps paradoxically, request people to speak up about whether they remained silent. Reports on safety voice are always at least one-step removed from the actual behaviour of raising or withholding safety concerns, are over-reliant on imagining or recalling behaviours, and cannot provide predictive insight into how safety voice relates to antecedents and outcomes. Accordingly, the validity of the research remains uncertain, and alternative methodologies focussing on actual behaviour are required to validate findings, and evidence interventions. The use of experiments in related domains (e.g., bystander intervention; P. Fischer et al., 2011) suggest these methods can provide a way to overcome the unique challenges of studying safety voice in hazardous situations. We thus propose that the shortfalls of safety voice methods (summarised in Table 1) can be overcome through the development of an experimental methodology that: (i) captures the behavioural nature of safety voice; (ii) avoids the reliance on memory and imagination; and (iii) explores the relationship to other variables as potential causes.

**Table 1:** Methodological shortfalls, needs, and experimental solutions for the investigation of safety voice.

**Shortfall**	**Need**	**Experimental solution**
**BEHAVIOURAL NATURE OF SAFETY VOICE**		
1. Reports provide no behavioural data	Reproduce safety voice behaviours	Create a situation to elicit speaking-up and silence
2. Few methods have operationalised safety voice as emerging from a clear hazard and safety concern	Operationalise a hazard that elicits a single safety concerns and behavioural response	Present a single hazard and ascertain safety concerns
3. Participants cannot be exposed to real hazards and cannot be aware their decisions on safety voice are observed	Minimise potential harm to participants while they believe risk is real	Manipulate the perception of risk, not real risk, using deception procedures
**THE RELIANCE ON MEMORY AND IMAGINATION**		
4. Reports provide inaccurate data on safety voice	Operationalise measures that directly observe safety voice behaviours	Record behaviours through observation (in-person/recording)
5. Floor and ceiling effects can bias estimates of behaviour	Provide measures that enable sufficient variance and observe speaking-up and silence	Calibrate safety concerns to elicit speaking-up and safety silence
**THE RELATIONSHIP WITH OTHER VARIABLES**		
6. Reports provide limited insights into causal relationship between safety voice and antecedents and outcomes	Provide methodologies that can establish and replicate causal relationships	Build a protocol that can manipulate variables of interest
7. Reports on safety voice may be subject to structural confounds introduced through sampling	Minimise the influence of unintended contextual confounds	Sample participants using random procedures
8. A third outcome variable is created if alternative mitigations are possible	Establish a method that limits alternative hazard mitigations to speaking-up and silence	Minimise alternative mitigations of the hazard
9. Relationships may not be reliable over time	Protocols need to enable direct replication and falsification	Provide a clearly specified study protocol

#### The Behavioural Nature of Safety Voice

Research on safety voice has emerged due to recognition that, in high-risk situations, raising concerns is critical to avoiding accidents. Case study investigations have revealed acts of raising and withholding of safety concerns as critical determinants of harm in dangerous situations (e.g., Moorhead et al., 1991; Cocklin, 2004), and the phenomenon is highly behavioural. It typically involves an individual (e.g., an employee, patient, concerned stakeholder) having a concern about a safety issue, and then raising it with another party (e.g., supervisor, doctor, colleagues) in order to prevent harm, or holding back from raising the concern altogether (silence). Yet, and despite the recognised importance of raising safety concerns for avoiding accidents (and silence in allowing accidents; Moorhead et al., 1991; Tarnow, 1999; Francis, 2013; Reader and O'Connor, 2014), investigations into this phenomenon have frequently assumed that reports correspond to real-world behaviour, and are subject to the same mechanisms that drive safety voice (Del Boca and Noll, 2000).

This is problematic because of: (i) the often-observed gaps between reports and actual behaviour (e.g., Sheeran, 2002); (ii) the lack of behavioural data upon which to base findings and interventions (Weathington et al., 2010); and (iii) the low fidelity of actions and context (i.e., operationalisations do not correspond to the behaviour and risky environment; Stoffregen et al., 2003). Accordingly, it remains unclear to what extent safety voice behaviours differ from report-based data and should be observed directly, in a standardised way (i.e., reports may not acquire behavioural data; Shortfall 1), or conceptualised, operationalised, and measured as emerging from clear hazards that cause safety concerns (i.e., safety concerns have not been measured alongside safety voice behaviours; Shortfall 2). Establishing this is important for generating accurate baseline data on safety voice (e.g., the average rates of people that are concerned about a hazard and speak-up or remain silent), clarifying the relationship between presented hazards and the extend that these cause concerns, and for generalising and predicting safety voice behaviours.

However, to date, 76% of the safety voice literature (Noort et al., submitted) has focussed on willingness to raise safety concerns in general (e.g., agreement to generic questionnaire items), post-intervention changes in safety voice, or the extent of safety voice in response to presented hazards without measuring safety concerns (yet for a safety concern item, see: Schwappach and Gehring, 2014c). For example, high-fidelity training simulations (e.g., Hanson, 2017) have specified safety voice as a trainable behaviour, whilst only measuring changes in safety voice in pre- and post-training questionnaires, and studies that have exposed participants to (perceived) hazards such as a senior person engaging in unsafe acts (e.g., Barzallo Salazar et al., 2014; Aubin and King, 2015) or medical emergencies (Reime et al., 2016) have assumed that such hazards should trigger safety concerns (yet do not measure this). Furthermore, where observational data on safety voice has been obtained, measurements have included safety voice into higher level codes (e.g., “team cooperation”; Hughes et al., 2014; Reime et al., 2016), focussed on a tendency to speak-up or remain silent without measuring safety concerns (Kolbe et al., 2012, 2014), assumed knowledge about hazards or their presentation elicited safety concerns (Barzallo Salazar et al., 2014), or presented multiple hazards at once (Hodges, 2018). To our knowledge, no studies have investigated the relationship between observed levels of safety voice and reported safety voice, or to measured safety concerns.

This is important, because safety voice is a highly contextualised behaviour: it is assumed to occur in response to the perception of safety being threatened within a particular context (e.g., cockpit, operating theatre, production line) that can be highly ambiguous (e.g., contrasting information, multiple hazards) and complex (e.g., March and Olsen, 1975). Without collecting data on perceptions of risk within a given context one cannot (i) compare across hazardous situations (e.g., threats to patient and aviation safety; Tamuz and Thomas, 2006) and (ii) make assumptions about why someone may have remained silent (i.e., unconcerned silence vs. withholding of safety concerns due to fear of reprisals), or (iv) ascertain whether voice occurred due to concern or precaution (i.e., unconcerned voice). Whilst self-report studies can provide insight on general tendencies for safety voice, insights on how safety concerns elicit safety voice behaviours remain minimal (cf. Schwappach and Gehring, 2014c), and behavioural studies have not measured the risk perceptions of the participants being observed.

To study similar phenomena in other fields, experimenters have designed standardised situations for eliciting participant behaviour: for example bystander interventions (for a meta-analysis see: P. Fischer et al., 2011) or defiance/resistance to authority (Milgram, 1963; Miller et al., 1995; Kaposi, 2017). Within the field of voice more generally, experiments have been used to investigate employee voice for volunteering non-safety related information (Morrison et al., 2015). What is common to these studies is that they create a high-fidelity illusion of an emerging problem that requires a behavioural response (e.g., helping a person falling victim to verbal abuse in a bystander scenario; P. Fischer et al., 2006) without endangering participants. Their benefit is that they allow for a behavioural phenomenon to be investigated in a highly controlled environment, with observations then being contextualised to specific scenarios.

To investigate safety voice, a similar approach would be beneficial, with participants engaging in standardised situations that create a safety concern that can be addressed through speaking-up. This is challenging because participants cannot be exposed to genuine physical harm and, to avoid observer effects and study naturalised behaviour (Nichols and Maner, 2008), participants should not be aware that their decisions on safety voice are being observed (Shortfall 3). These issues can only be addressed through designing scenarios that manipulate *perceived* levels of safety (i.e., hazards that elicit a concern, and a need to intervene), not actual risks, while measuring safety concerns and ensuring participant remain naïve to study goals through deception procedures (Weathington et al., 2010). In particular, designing plausible cover stories is important: in the absence of these invalid data may emerge because participants (i) deduce the hazard is fabricated; or (ii) believe (correctly) that researchers would need to comply with ethical standards that would prevent the scenario.

In summary, an experimental paradigm is required to investigate safety voice in a controlled, standardised, and generalisable way. A key property of any such paradigm is that it elicits observable safety voice behaviours (i.e., both raising and withholding concerns) through manipulating perceived risk and ascertaining safety concerns (i.e., as opposed to exposure to real physical harm), with deception procedures ensuring that participants are naïve to study intentions, and thus their behaviour is natural.

#### The Reliance on Memory and Imagination

Insight on safety voice has largely been generated through recalled or imagined (in)action during hazardous instances. Whilst practice-based inquiries have investigated actual incidents (Rogers, 1986; Francis, 2013), typically, it is assumed that participants are accurate in remembering and generalising past behaviours (e.g., Schwappach and Gehring, 2014d), or can imagine how they would respond in a safety-related situation (Schwappach and Gehring, 2014c). These data are then used to explain the factors that influence safety voice (e.g., Nembhard et al., 2015), to describe its occurrence (Tucker et al., 2008), and predict future outcomes (Blanco et al., 2009). Yet, the validity of this approach is not self-evident, and correlations often low (Reiss, 1971), with participants in report-based studies having been long-shown as unable, or unwilling, to provide accurate data (Bartlett, 1932; Podsakoff and Organ, 1986). Memories are influenced by a limited ability to recall situations: behaviour can be activated by causes outside of conscious awareness at the time of the behaviour such as scents, posters or semantic primes (Aarts et al., 2008; Custers and Aarts, 2010). Furthermore, distances in time, space, or person (e.g., CEOs reporting whether staff in remote locations raised safety concerns for a system introduced the previous year) can further erode data accuracy, and recalling and imagining behaviours is subject to subject-matter expertise and cognitive biases (e.g., availability heuristic; Schwarz et al., 1991). That is, participants may lack knowledge on what constitutes speaking-up, or be unwilling to accurately report safety silence: reports are constructed based on individual attitudes and perceived social norms regarding safety voice (Bartlett, 1932); individuals may experience dissonance between their ideal self-image as able to speak-up and admitting to safety silence (Baumeister, 1982); and social desirability biases half of survey and interview findings (van de Mortel, 2008). For example, desires to appear a good and ethical employee (or effective manager), may bias participants toward reporting speaking-up over safety silence, especially when harmful outcomes occurred in serious or obvious safety situations.

Moreover, recalling and imagining safety voice provides limited scope for exploring the dynamic context in which it occurs. Safety voice surveys, interviews, and vignettes typically aim to increase realism through recall of previously experienced hazardous scenarios or the presentation of scenarios validated by subject matter experts. However, these scenarios remain limited because (i) the hazard environments they present will usually differ in some way from reality (i.e., which hinders recall; Schwabe and Wolf, 2009); (ii) the dynamic of a situation is not present (e.g., task pressures on the participant); and (iii) there are no immediate consequences for participants, or safety, at the time of data collection. This means, as shown in other research paradigms (Milgram, 1974; Blass, 1999), a gap may exist between reports and behaviour, and addressing this is important for establishing the triggers of safety voice (e.g., hazard perception), and the contextual factors (e.g., interactions between people and situations) that determine voice: or, indeed, silence.

The above factors potentially erode the accuracy of safety voice data collected through report-based methods (Shortfall 4), which undermines the validity of conclusions assumed from data (Bagozzi et al., 1991), and more specifically, how safety voice is assumed to be operationalised in risky situations. Addressing this is important for establishing the triggers of safety voice, and the contextual factors (e.g., interactions between people and situations) that determine the behaviour. An experimental paradigm focussed on eliciting safety voice can address this limitation through facilitating observations of safety voice (e.g., at the time of data collection, or through video), ensuring these are reliably assessed (e.g., using inter-coder reliability for the extent to which an individual raised safety concerns), with participant *post-hoc* reports being matched to behavioural data. To achieve this, and undertake meaningful statistical analyses, safety voice experiments need to elicit both safety voice acts (i.e., raising a concern) and silence. Floor (i.e., near-complete silence) and ceiling effects (i.e., near-complete voice) can bias estimates of the behaviour (Shortfall 5), with information about change (e.g., through interventions) being lost at the extreme ends of the scale through data censoring (i.e., relevant data falling beyond the scale end-point; Cox and Oakes, 1984). Though statistical procedures are available (McBee, 2010), a successful experimental paradigm should produce sufficient statistical variance and a moderate degree of speaking-up and silence (i.e., a 50–50 split). Thus, an experimental approach enables direct observations of safety voice behaviours, and provides scope for statistical analyses that can evidence higher construct validity.

#### The Relationship With Other Variables

Data collection using report-based methodologies typically collect data on safety voice and other variables simultaneously (e.g., in the same survey), and using populations that are not randomised. This limits interpretation of the factors that determine or follow safety voice and silence behaviours.

Investigations using reports provide limited insights into causal relationship between safety voice and antecedents and outcomes (Shortfall 6). Yet, to build interventions, safety voice measures need to establish and replicate causal relationships. Antecedents and outcomes have been linked with safety voice and silence, and evidence suggests that interventions can successfully alter reported levels of safety voice. For example, safety silence increases with perceived social risks (e.g., ramifications of speaking up; Bickhoff et al., 2016), differences in safety knowledge (e.g., Schwappach and Gehring, 2014b), hierarchical power relations (e.g., Seiden et al., 2006), and, conversely, training on why and how to speak up reduces silence (Johnson and Kimsey, 2012; Delisle et al., 2016; Kulig and Blanchard, 2016; Hanson, 2017). Yet, such observations tend to be correlational rather than causal in nature. Additionally, controlled manipulations of safety voice antecedents through vignettes (Schwappach and Gehring, 2014c; Anicich et al., 2015; Aubin and King, 2015) or interventions (Habyarimana and Jack, 2011; Hanson, 2017) are scarce and tend to rely on indirect data rather than behavioural observations.

Furthermore, reports on safety voice may be subject to structural confounds (i.e., variables that are not of interest but covary with independent variables and provide alternative explanations of results; Goodwin, 2008) that may emerge from contextual variables that are introduced through sampling (Shortfall 7; e.g., junior doctors needing longer to accrue subject-matter expertise in part of the included research contexts). To establish valid conclusions, measures need to minimise the influence of confounds and minimise alternative explanations of relationships between antecedents and safety voice and silence. Yet, report-based methodologies have sampled within similar populations (e.g., oncology departments, medical students; Schwappach and Gehring, 2014a; Delisle et al., 2016), and across different populations (e.g., healthcare, construction, retail; Manapragada and Bruk-lee, 2016), and both sampling practices can be problematic because unmeasured and uncontrolled characteristics of contexts (e.g., workload; Nembhard et al., 2015) can provide alternative explanations of patterns in safety voice. Addressing this is important, and a need exists to minimise the influence of unwanted contextual confounds through applying random sampling procedures.

Hence, a need remains to establish methodologies that can address the relationships between safety voice and other variables. The optimal way to achieve this is through safety voice experiments. These can manipulate antecedents (i.e., enabling causal conclusions), randomise participants (i.e., randomising confounds across the groups to eliminate structural influences), and limit participants' influence on hazard mitigation to a choice on whether to speak up. Critical to an experimental paradigm is that participants should not be able to mitigate physical harm through other means than speaking-up: a third outcome variable is created when alternative mitigations are possible (Shortfall 8). This means that, when participants have a safety concern, safety silence can be determined through absence of safety voice. The field experiment by Barzallo Salazar and colleagues (Barzallo Salazar et al., 2014) showed how surgeon communication style predicts medical students' tendency to speak up, yet the field experiment did not assesses safety concerns and thus cannot distinguish concerned and unconcerned silence, and because relationships between psychological variables may not be reliable over time (Shortfall 9; Gergen, 1973) a need remains for available experimental protocols that enable the direct replication and falsification of findings (Earp and Trafimow, 2015) in laboratory settings.

### The Current Article

We propose the first experimental paradigm for investigating safety voice in laboratory environments, and establish and evaluate it across three studies in order ensure the protocol meets the nine requirements reported in Table 1 that address the shortfalls of current safety voice methodologies. Through doing this, we aim to advance safety voice research by (i) enabling a behavioural approach, (ii) moving away from a reliance on memory and imagination, and (iii) supporting the investigation of causal relationships between safety voice and other variables, which can be used as a basis for intervention.

Below, we describe the “Walking the plank” paradigm that we have developed for investigating safety voice. We then report on the three studies used to refine and iterate the paradigm, alongside the observations about safety voice yielded from these studies.

#### The “Walking the Plank” Paradigm

Our proposed paradigm for assessing safety voice, the “Walking the plank paradigm” introduces a decision-point for participants in which they are faced with a hazard (a plank with the potential to break when walked on), and need to decide to either raise their safety concern (and experience any consequences of safety voice) or remain silent and let the situation run its course (with potential harmful implications for victims of the hazard). The paradigm's title is a reference to the naval practice of coercing victims to walk off a plank, plunging into the open sea and certain doom. The parallel is in the fact that perpetrators felt abdicated of responsibility because the victim ostensibly killed themselves (i.e., for onlookers, it was an act of safety silence rather than murder). Our Walking the plank paradigm is generic, and its realistic perceived consequences and randomisation of participants provide for a confound-free assessment of safety voice that enables generalisable conclusions. Before settling on a viable scenario, we considered and abandoned four hazardous scenarios for the experimental investigation of safety voice: crossing a busy road (i.e., the real risk was considerable), faking a terrorist threat (i.e., too politically sensitive; likely to upset participants), interacting with loose electric wiring (i.e., the hazard could be mitigated by the participant through alternative means than safety voice such as unplugging the equipment), and ordering participants to provide approval for future hazardous experiments (i.e., difficult to ascertain risk perceptions; no immediate consequences at time of data collection).

The final scenario involved a person walking across a plank with a perceived low weight limit in the context of an alleged creativity task (the cover story). We chose this hazard because we could manipulate the perception that the plank might break (by having a bendy plank and stating a weight limit) while using a plank that was actually safe. Furthermore, it enables experimental control of variables of interest (e.g., self or other walking on the plank), safety knowledge (i.e., provided information regarding the maximum load of the plank), a plausible cover story (i.e., participation in a creativity task to evaluate and test creative uses of wooden materials), evaluative mindsets (i.e., participants evaluated aspects of the task), standardisation of the hazard (i.e., consistent materials and research assistants), testing of risk perceptions and safety concerns (i.e., perceived maximum load of the plank and the person sitting/walking on it), a straightforward and resource efficient replication by others, and a systematic observation of the linguistic nature of safety voice (this is beyond the scope of the current article). In this article we show that this paradigm meets our nine criteria.

To test the scenario, we iterated it across three studies. Our goal was for the paradigm to meet the nine requirements (see Tables 1, 2) of an effective safety voice experiment. Demonstrating and reporting on this process is important for (i) enabling the effective application of the Walking the plank paradigm (e.g., it highlights potential challenges for future research), (ii) supporting open science (i.e., protocol histories enable more direct replication; it acknowledges safety voice experiments are challenging and that the final version emerged from addressing this) and (iii) supporting future research on safety voice (i.e., it illustrates how amendments to the paradigm can be made and evaluated).

**Table 2:** Protocol characteristics of study 1, 2, and 3.

**Protocol characteristic**	**Study 1**	**Study 2**	**Study 3**
**HAZARD**			
Hazard	Sitting	Sitting	Walking
Plank material	Pinewood	Plywood	Plywood
Stated maximum load of plank	45 kg	42 kg	30 kg
Presence of broken plank	Yes	Yes	No
Implicit risk condition included	Yes	No	No
Victim of hazard	Condition (Participant/RA: ns)	RA	RA
Ideas evaluated by participant	Seesaw, Shelving, Door, Juggling, Chair/bench, Slide	Shelving, Mirror, Juggling, Bench, Piece of art	Shelving, Mirror, Juggling, Footbridge, Piece of art
Risk perception calculated	Yes (participants' weight)	Yes (estimated RA's weight)	Yes (estimated RA's weight)
Reported safety concerns in wrap-up questionnaire	No	Yes	Yes
**SAFETY VOICE**			
Direct observation of safety voice behaviours	Yes	Yes	Yes
Observation of safety silence behaviours	No	Yes	Yes
Reported safety voice in wrap-up questionnaire	No	Yes	Yes
**RESEARCH ASSISTANT (RA) ACTIONS**			
RA avoids hazard upon safety voice	Not manipulated	Yes	Yes
RA perceived to be naïve to maximum load	Not manipulated	Yes	Condition (yes/no: ns)
RA indicates to respond negatively to speaking-up	Neutral	Condition (yes/no: ns)	Neutral
Perspective taking with RA	Not manipulated	Not manipulated	Condition (be objective/imagine self as other: ns)
**QUESTIONNAIRES**			
Wrap-up questionnaire	Yes	Yes	Yes
Demographic questionnaire (submitted before study)	Yes	Yes	Yes

*ns - effect of the manipulation was not significant*.

Through the course of three studies (their characteristics are summarised in Table 2), we illustrate that the Walking the plank paradigm meets the requirements for safety voice and silence experiments. In brief, in study 1 we demonstrate that the paradigm can elicit safety voice behaviours in a safe, controlled and randomised laboratory environment. In study 2 we refine the protocol and demonstrate it is possible to elicit safety silence. In study 3 we further refine the protocol to enable sufficient risk perceptions and explore the nature of safety voice behaviours.

## Study 1

The aim of study 1 was to establish the protocol for the Walking the plank paradigm (initially “sitting on the plank”), and provide a first evaluation. Within the guise of a creativity task, participants experienced a perceived hazard designed to elicit safety voice behaviours (i.e., being asked to sit on a plank with a risk of breaking under heavy load). The goals of study 1 were to (i) test whether the paradigm could sufficiently elicit safety voice behaviours in response to potential physical harm from breaking the plank; (ii) present a perceived, not actual hazard; (iii) observe safety voice directly; (iv) apply participant randomisation and deception procedures; and (v) introduce the experimental manipulation of variables (i.e., minimising harm, hazard presentation, hazard awareness, deception, victim identity) for determining safety voice.

### Method

#### Protocol

A 2(safety: unsafe-control) ^*^ 2(victim: participant-research assistant) design was employed. Participants were invited to a study about “creativity” and allocated to study conditions using double blind and random procedures. The study consisted of three stages. First, participants completed a 5-min “creativity task” in which they had to design creative uses of a pinewood plank (L: 120 cm, W: 20 cm, H:1.8 cm) and four blocks of wood. The instruction read: “In this room you find a plank and four pieces of wood. In the box below, write down how you could use a plank and four pieces of wood. Try to be creative and think of as many solutions as you can. You have 5 min.” Second, in an interaction with a research assistant, the participants were instructed to undertake and rate the feasibility and creativity of each idea, but were informed that they would test the previous participant's ideas (a standard set: seesaw, shelving, door, juggling, chair/bench, slide) which included a hazardous idea (i.e., “chair/bench”). Upon re-entering the room, the research assistant stated: “The next stage involves testing these ideas for two things: feasibility and creativity. However, your ideas will be tested by the next participant, and now the ideas of the previous participant are tested.” Finally, participants completed an electronic questionnaire (including manipulation checks for hazard awareness and naivety to study hypotheses, and unpresented exploratory variables), after which they received a full debrief.

To present the hazard, and elicit a behavioural response, the instruction for the creativity task included a note on the maximum load of the plank (i.e., “Please note: the plank can carry a maximum load of 45 kg/99 lbs/7.1 stone)”; (unsafe condition), or no additional note (control condition). Furthermore, a broken version of the plank in the room reinforced this information. In reality, the plank was able to hold at least 125 kg. When testing the previous participant's creative ideas, the participant was prompted by the research assistant to place the plank across two chairs (their location marked discretely on the floor) with a gap for a third chair between them. The research assistant then made clear their intention to test the feasibility of the bench through sitting on it (e.g., “Okay, let me test this”) or requested the participant to sit (e.g., “Could you please demonstrate?”). The emphasis of the protocol was to observe any subsequent speaking-up or silence behaviour. The protocol concluded with the participant completing a questionnaire.

Ethical approval was obtained for all studies from LSE's research ethics committee (#000540), and informed consent was required from participants before commencing. To comply with data regulations, anonymous data storage to enable future research was included as a separate question.

#### Participants

129 participants (N_females_ = 85, N_students_ = 98) were recruited from a pool including students and the general public. Participants were spread in age [M_(sd)_ = 26.57_(7.56)_] and weight [M_(sd)_ = 64.81 kg_(14.41)_]. On a 5-point Likert scale (with 1 = low), participants indicated they had no expertise on timber [M_(sd)_ = 1.67_(1.03)_], or whistleblowing legislation [M_(sd)_ = 1.48_(0.83)_], and safety voice did not correlate with demographic variables (i.e., student status, gender, age, social economic status, class, education, expertise on timber/whistleblowing, nationality, language). One participant was dropped from analyses because the protocol was not followed.

#### Measures

##### Manipulation checks

A perceived risk was calculated from two items in the questionnaire that followed the scenario (i.e., kilograms of participants' own weight minus the estimated plank's maximum load). This measure addressed that the plank's maximum load would not pose a safety issue without a person sitting on it. One participant's estimation of the maximum load of the plank (i.e., 292 kg) was removed based on a Cook's test identifying the response as an outlier (i.e., for the effect of the safety condition on risk perception; Cook = 0.50). The questionnaire asked whether participants noticed anything odd during the study.

##### Safety voice

A direct observational measure of safety voice was used. Safety voice (1) was coded if the participant questioned whether testing the bench was a good idea and/or alternative action might be more appropriate (e.g., “Did the instruction not state a maximum of 45 kg?”; “This would be feasible for a child, not for adults”), before the chair/bench was tested. Otherwise the participant's behaviour was recorded as “no voice” (0). Through discussing examples, research assistants were trained to recognise whether statements intended to prevent a situation in which someone sat on the plank and might break it. The first author made a final decision through watching video recordings when research assistants were unsure on how to code participants statements.

##### Prohibitive employee voice

Three items from Liang et al. (2012) were adapted to the laboratory environment to explore overlap with safety voice (on 5-point Likert scale, with 5 indicating strong agreement): “I pointed out problems when they appeared, even if that would hamper relationships with others”; “I advised others against undesirable behaviours that might hamper the task”; “I highlighted problems that might cause serious issues.”

### Results

#### Manipulation Check

The paradigm's safety manipulation created a perception that sitting on the plank would break it (i.e., weight difference between person sitting and plank's maximum load ≥0 kg). The perceived maximum load of the plank was 13.96 kg lower in the unsafe condition [M_(se)_ = 48.84 kg_(2.97)_], *F*_(1, 127)_ = 4.39, *p* = 0.04, η^2^ = 0.03, *observed power* = 0.55. The perceived risk for the unsafe condition [M_(se)_ = 19.60 kg_(3.26)_] was non-zero, *t*_(59)_ = 6.00, *p* < 0.001, higher than the control condition [M_(se)_ = −1.32 kg_(5.97)_], *F*_(1, 127)_ = 7.72, *p* = 0.006, η^2^ = 0.06, *observed power* = 0.79, and led 81% of participants in the unsafe condition (95CI: 71–91%) to think the plank would break, *t*_(61)_ = 15.94, *p* < 0.001. Illustrating successful deception, no participant guessed the true nature of the study.

#### Safety Voice

The safety manipulation successfully elicited safety voice. Whilst some participants raised safety concerns in the control condition (i.e., 20% spoke up; 95CI: 10–29%), *t*_(66)_ = 3.99, *p* < 0.001, participants were 2.76 times more likely to raise safety concerns against sitting on the bench when information regarding an unsafe maximum load was provided, *Wald*(1) = 6.12, *p* = 0.01. Yet, and despite the success of the manipulation to create risk perceptions for 81% of participants in the unsafe condition, a considerable proportion of participants in the unsafe condition did not raise a concern (60%; 95CI: 48–73%), and this held when participants without a perceived risk were accounted for: 58% (95CI: 44–72%) remained silent about their perceived risk (see Table 3). Furthermore, in the unsafe condition, 33% (95CI: 2–65%) of participants raised a safety concern despite not perceiving a risk, *t*_(11)_ = 2.35, *p* = 0.04, and perceiving risk was not related to safety voice, χ^2^_(1)_ = 0.30, *p* = 0.58. However, whilst the safety manipulation caused differences in safety voice, no influence was found on prohibitive employee voice, *F*_(1, 127)_s < 1.29, *p*s > 0.26, and no correlation existed with observed safety voice, *r*s < |−0.10|, *p*s > 0.25. This suggests that hazards differentiate safety voice but the relationship between risk perception and safety voice is not straightforward. A need thus exists for improved safety concern measures. Finally, the identity of the victim (i.e., participant vs. research assistant) did not influence safety voice, *ns*^1^.

**Table 3:** Safety voice behaviours for Study 1 (unsafe condition).

	**Perceived risk**		**Perceived no risk**		**Total**	
	**N**	**%_**(SE)**_**	***n***	**%_**(SE)**_**	***n***	**%_**(SE)**_**
Voice	21	42_(7.1)_	4	33_(14.2)_	25	40_(6.2)_
Silence	29	58_(7.1)_	8	67_(14.2)_	37	60_(6.2)_
Total	50	81_(5.1)_	12	19_(5.1)_	62	100_(−)_

*Percentages total 100% within a column, except for the total of perceiving (no) risk*.

### Discussion

Study 1 demonstrated that the paradigm enables (i) the reproduction of safety voice behaviours in response to a hazard (speaking-up only); (ii) the presentation of a perceived, not actual, hazard; (iii) the direct observations of safety voice; (iv) participant randomisation to minimise alternative explanations; and (v) experimental control over study variables (i.e., minimising harm, hazard presentation, hazard awareness, deception, victim identity). Furthermore, it suggested that the relationship between risk perceptions and safety voice is not straightforward, and participants can remain silent when perceiving a risk, or speak-up when not perceiving a risk.

However, study 1 did not fully illustrate five requirements for safety voice experiments. First, participants raised safety concerns when demonstrating the seesaw and slide ideas, thus presenting multiple hazards and potentially producing unmeasured spillover effects. Second, it was not clear whether the perception of risk made people concerned about the hazard: it is not self-evident that safety concerns emerge from participants' body weight, or that the application of this weight to a plank with a low capacity always leads to concerns, and in order to demonstrate safety silence (i.e., the withholding of safety concerns) experiments need to establish optimal measures to establish safety concerns. This is important, because, third, whilst safety voice behaviours were observed, these emerged for people with and without perceptions of the plank potentially breaking, and in the absence of clear safety concern measures it is unclear whether a lack of voice meant safety concerns were withheld (i.e., participants might not have been concerned about harm despite a perceived likelihood of the plank breaking). Fourth, the proportion of safety voice acts was low and could be improved to prevent floor effects. Finally, when participants were victim, they occasionally mitigated the hazard by keeping weight on their feet and thus not fully sitting on the plank (creating a third outcome variable).

## Study 2

Study 2 aimed to address the issues raised in study 1 through amending the risk perception measures to enable the observation of safety silence (i.e., calculated based on the person sitting on the bench and triangulated with an item on having a safety concern); eradicating safety voice for multiple hazards; improving the manipulability of the perceived physical risk to elicit stronger responses (i.e., lowering the weight limit; using a bendy plank; creating sufficient variance in safety voice and silence); and minimising alternative ways to mitigate physical harm following from breaking the plank^2^.

### Methods

#### Protocol Refinements

The protocol in study 1 was followed, albeit with five adjustments. First, the observation of safety silence was enabled through an altered risk perception measure and self-report safety voice questionnaire item to obtain additional data and ascertain whether the scenario led to subjective safety concerns. Second, to increase the perceived risk of physical harm, the maximum load was lowered slightly to 42 kg (93 lbs, 6.6 stone) and the pinewood plank was replaced by a more bendy plywood plank of the same proportions (still capable to withstand at least 125 kg in reality). Third, to eliminate other perceived hazards from the protocol, three ideas (i.e., seesaw, door, slide) were replaced with two new ideas (i.e., mirror, piece of art). Fourth, to ensure that the hazard could not be mitigated through not fully sitting on the plank, the research assistant sat on the plank. Finally, based on a pilot study, only the unsafe scenario was included^3^.

#### Participants

Sixty-nine participants were recruited [N_females_ = 50; N_students_ = 62; Age M_(sd)_ = 25.52_(0.61)_]. Participants had no expertise on timber [M_(sd)_ = 1.54_(0.11)_], or whistleblowing legislation [M_(sd)_ = 1.62_(0.12)_], and demographic variables were not associated with safety voice measures.

#### Measures

##### Manipulation check

Perceived risk was based on the estimated weight of the research assistant (i.e., estimated weight of the research assistant's above the plank's maximum load). Furthermore, a dichotomous item asked whether participants were concerned regarding the demonstration of the bench (answered as: yes/no). One participant's estimation of the maximum load of the plank (i.e., 200 kg) was removed based on a Cook's test identifying the response as an outlier (i.e., Cook = 0.09).

##### Safety voice

Safety voice acts were observed as a dichotomous variable, described in study 1. Furthermore, participants' self-reported safety voice was measured as a dichotomous variable (i.e., did you raise a safety concern regarding the demonstration of the bench idea: yes/no). Safety silence was operationalised as participants who said they held a safety concern but were not observed to raise it.

### Results

#### Manipulation Check

The safety manipulation created a perception that sitting on the plank could break it (i.e., excess weight ≥ 0 kg): the perceived maximum load of the plank [M_(se)_ = 60.28 kg_(3.20)_] was not statistically higher than the weight of the research assistant sitting on the plank [M_(se)_ = 57.69 kg_(1.26)_], *t*_(67)_ = −0.87, *p* = 0.39, 55% of participants (95CI: 43–67%) perceived that the plank could break, *t*_(66)_ = 9.02, *p* < 0.001, and 42% (95CI: 30–54%) reported feeling concerned, *t*_(68)_ = 7.02, *p* < 0.001. The new safety concern measure had a stronger relationship to safety voice than perceived risk: whether participants perceived a physical risk was not related to observed safety voice behaviours, *OR* = 1.64, *Wald*(1) = 0.79, *p* = 0.37, yet whether participants reported having a safety concern about the act of sitting on the plank related to safety voice, χ^2^_(1)_ = 4.14, *p* = 0.04, and these people were 3.16 times more likely to be observed to raise a safety concern, *Wald*(1) = 3.81, *p* = 0.05. Furthermore, safety concerns were predicted by the perceived risk, *OR* = 3.61, *Wald*(1) = 5.87, *p* = 0.02, indicating an indirect relationship between perceived risk and safety voice behaviours through safety concerns.

#### Safety Voice

Replicating study 1, safety voice behaviours were directly observed, but 71% (95CI: 59–82%) of participants did not raise a concern about the research assistant testing the bench, *t*_(67)_ = 5.28, *p* < 0.001. Yet, strikingly, and demonstrating concerned silence, this held when participants without safety concerns were not included in the analysis: 57% of participants did not raise their safety concern (95CI: 38–76%), *t*_(27)_ = 4.50, *p* < 0.001. Furthermore, and suggesting the existence of two additional types of safety voice behaviours (i.e., unconcerned voice and silence), 20% (95CI: 7–33%) of participants raised a safety concern despite being unconcerned, *t*_(39)_ = 3.12, *p* = 0.003 (see Table 4).

**Table 4:** Safety voice behaviours for study 2.

	**Concerned**		**Unconcerned**		**Total**	
	***n***	**%_**(SE)**_**	***n***	**%_**(SE)**_**	***n***	**%_**(SE)**_**
Voice	12	43_(9.5)_	8	20_(6.4)_	20	29_(5.5)_
Silence	16	57_(9.5)_	32	80_(6.4)_	48	71_(5.5)_
Total	28	42_(6.0)_	40	58_(6.0)_	68	100_(−)_

*Percentages total 100% within a column, except for the total of (un)concerned. (Missing: 1)*.

#### Self-Reported Safety Voice

Supporting the need for direct behavioural observations of safety voice behaviours, participants provided poor report of their behaviours. Reported safety voice related to observed safety voice (*r* = 0.47, χ^2^_(1)_ = 14.94, *p*s < 0.001), but it only explained a small proportion of the variance (*R*^2^ = 0.22), and 23% of participants (95CI: 13–34%) misreported their behaviour, *t*_(67)_ = 14.76, *p* < 0.001, with participants 4.5 times more likely to misreport that they raised a concern, *Wald*(1) = 6.25, *p* = 0.01.

### Discussion

Study 2 successfully addressed the requirements to observe safety silence, ascertain safety concerns for a single hazard, and minimise alternative hazard mitigations. Furthermore, study 2 showed it is important to include safety concern measures in safety voice experiments, indicated that safety voice consists of four behaviours (i.e., concerned voice and silence; unconcerned voice and silence), demonstrated a gap between observed and reported safety voice, and indicated that participants tend to misreport in favour of speaking-up.

However, study 2 was limited in terms of eliciting safety concerns from the majority of participants, with even fewer participants (as a proportion of concerned participant) raising their concern. The reasons for this are unclear, yet consistent with the wider safety voice literature, may reflect either an unwillingness to voice safety concerns, or a perception that the situation does not merit action (unconcerned silence). In particular, only 42% of participants were concerned about the act of sitting on the plank (and of these 57% withheld their concern). This indicates that for the majority of participants the task was not particularly risky, and for those who did perceive it as risky, it may not have been perceived as sufficiently dangerous to warrant intervention. Thus, to increase engagement in safety voice behaviours, and prevent a floor effect, we decided to further increase participants concern.

## Study 3

Study 3 refined the paradigm so that it would meet the final requirements for a safety voice paradigm: to increase the number of participants with safety concerns and produce an equal amount of safety voice and silence acts. It replicated the four safety voice behaviours identified in study 2, and refined the protocol through further reducing the stated maximum load of the plank to 30 kg and altering the interaction with the plank to walking the plank, rather than sitting^4^.

### Methods

#### Protocol Refinements

To improve the number of concerned participants, study 3 refined the paradigm's protocol through replacing the previous participant's idea for creating a bench by a footbridge. Instead of sitting on the plank when it is placed across two chairs, the research assistant made clear he/she would be testing the idea by walking over it (and did so in the absence of safety voice)^5^. The final protocol is presented in an online manual (providing detailed pictures, scripts)^6^.

#### Participants

Seventy-five participants were recruited [N_females_ = 49; N_students_ = 69; Age M_(sd)_ = 23.09_(3.87)_, missing demographic data: 1 person]. In reply to dichotomous questions (i.e., are you an expert on wood/whistleblowing), no participants reported to be whistleblowing experts and only 1 participant reported to be a wood expert. Demographic variables were not associated with safety voice measures.

#### Measures

Study 3 adopted the manipulations checks (i.e., perceived risk, self-reported safety concern) and safety voice measures (i.e., observed acts, self-reported) described in study 2. Ten additional exploratory items (on a 5-point Likert scale, with 5 indicating strong agreement) were included: “I felt I might be seen as a trouble-maker when I spoke up” (Wei et al., 2015); “I felt obligated to raise any concerns I had” (Liang et al., 2012); “Right now, I worry about making mistakes” (Carver and White, 1994); “I felt I might offend the RA by questioning the way things were done”; “I felt the RA might bring out the worst in me”; “I felt uncomfortable to speak up about concerns I had”; “I had a concern about something that I thought the RA was not aware of”; “I had more information than the RA”; “I withheld my opinions”; “I don't feel very sorry for any problems the research assistant might have” (reverse-coded).

### Results

#### Manipulation Check

Altering the safety manipulation to walking the plank improved concerns that the plank would break it (i.e., weight difference ≥ 0 kg): the perceived maximum load of the plank [M_(se)_ = 38.89 kg_(2.93)_] was significantly lower than the perceived weight of the research assistant walking the plank [M_(se)_ = 56.76 kg_(1.48)_], *t*_(73)_ = 6.08, *p* < 0.001, 82% of participants (95CI: 74–91%) perceived the plank could break, *t*_(73)_ = 18.51, *p* < 0.001, and 68% (95CI: 57–79%) reported feeling concerned, *t*_(73)_ = 12.54, *p* < 0.001. This proportion of concerned participants was significantly higher than for Study 2 (i.e., 42% concerned participants), *t*_(74)_ = 4.80, *p* < 0.001.

*Safety voice*. The protocol for Study 3 elicited speaking-up for 44% (95CI: 33–56%) of participants, *t*_(74)_ = 7.63, *p* < 0.001, and, demonstrating safety silence, this held when unconcerned participants were excluded, *t*_(50)_ = 7.21, *p* < 0.001: participants with safety concerns were split between those who raised (i.e., 51%) or withheld (i.e., 49%) their safety concerns, and, providing sufficient variation in safety voice and silence, this split was not different from a 50–50% split, *t*_(50)_ = 0.14, *p* = 0.89, and this was true for concerned and unconcerned participants, χ^2^_(1)_ = 3.15, *p* = 0.08. Furthermore, and providing further support for the existence of (un)concerned voice and silence, 29% of participants (95CI: 10–49%) raised a safety concern despite being unconcerned, *t*_(39)_ = 3.08, *p* = 0.01 (see Table 5).

**Table 5:** Safety voice behaviours for Study 3.

	**Concerned**		**Unconcerned**		**Total**	
	***n***	**%_**(SE)**_**	***n***	**%_**(SE)**_**	***n***	**%_**(SE)**_**
Voice	26	51_(7.1)_	7	29_(9.5)_	33	44_(5.8)_
Silence	25	49_(7.1)_	17	71_(9.5)_	42	66_(5.8)_
Total	51	68_(5.4)_	24	32_(5.4)_	75	100_(−)_

*Percentages total 100% within a column, except for the total of (un)concerned*.

A MANOVA (using Pillai's trace) suggested that participants who displayed either concerned voice, concerned silence, unconcerned voice or unconcerned silence responded differently to ten exploratory questionnaire items, *V* = 0.86, *F*_(30, 192)_ = 2.56, *p* < 0.001 η^2^ = 0.29, *observed power* = 0.91, and separate ANOVAs confirmed this, *Fs*_(3, 71)_ ≥ 3.41, *p*s ≤ 0.02, η^2^*s* ≥ 0.13, *observed power* ≥ 0.75. *Post-hoc* analyses suggested that people who raised their concerns were less fearful, more caring and thought they had more information compared to those who withheld their concerns: they were less likely to fear being seen as a trouble-maker, *MD* = −0.79, *p* = 0.01, offend the RA, *MD* = −0.79, *p* = 0.05, or making mistakes, *MD* = −0.86, *p* = 0.01, state to withhold their opinions, *MD* = −0.92, *p* = 0.01, feel sorry for any problem the research assistant had, *MD* = −0.77, *p* = 0.02, or obligated to raise concerns, *MD* = −0.81, *p* = 0.02, and think they had more information than the RA, *MD* = −0.81, *p* = 0.03. Furthermore, and suggesting a lack of safety concerns might be due to feeling less responsible for the research assistant, in comparison to those who raised their concerns, those who spoke-up despite being unconcerned felt less obligated to raise concerns, *MD* = −1.48, *p* = 0.004, and less sorry for the research assistant's problems, *MD* = −1.16, *p* = 0.02, and a marginally significant trend suggested they might have less concerns that they think the research assistant was not aware of, *MD* = −0.1.24, *p* = 0.06. Providing further evidence that concerned participants who remained silent were fearful, they were more likely than those who raised concerns despite being unconcerned to state they withheld their opinions, *MD* = 0.1.27, *p* = 0.03, and feel uncomfortable to speak up, *MD* = 0.1.15, *p* = 0.04. Finally, and suggesting that making people concerned can improve speaking-up when people display unconcerned silence, those who displayed unconcerned silence were more likely than those displaying unconcerned voice to feel obligated to raise concerns, *MD* = −0.1.24, *p* = 0.04, and less likely than those who raised concerns to perceive a concern that they felt the research assistant was not aware of, *MD* = −0.1.03, *p* = 0.02.

#### Self-Reported Safety Voice

Replicating Study 2, and supporting the need for direct behavioural observations of safety voice, participants provided poor self-reports of their safety voice behaviours. Self-reported safety voice for Study 3 related stronger to observed safety voice than for Study 2, *r* = 0.64, $\chi_{\left(1\right)}^{2}$ = 30.39, *p*s < 0.001. However, a considerable portion of the variance remained unexplained (*R*^2^ = 0.41), and 19% of participants (95CI: 10–28%) misreported their behaviour, *t*_(74)_ = −4.12, *p* < 0.001, but participants' tendency to misreport safety voice acts over safety silence was only a marginal trend, *OR* = 2.78, *Wald*(1) = 2.48, *p* = 0.09 (bootstrap sample = 1,000).

### Discussion

Study 3 successfully addressed the remaining challenges to the paradigm (i.e., creating sufficient safety concerns; producing equal safety voice and silence) through altering the presented hazard from sitting on the plank to walking over it. This amendment increased the number of concerned participants and thus creates ample scope to test interventions for safety voice because the resulting proportion of safety voice (i.e., 50%) could be improved and reduced through the manipulation of safety voice antecedents. Progress of the development of the Walking the plank paradigm across the 3 studies is summarised in Table 6.

**Table 6:** The illustration of requirements for safety voice experiments across study 1, 2, and 3.

**Requirement**	**Study 1**	**Study 2**	**Study 3**
**BEHAVIOURAL NATURE OF SAFETY VOICE**			
1. Created a situation to elicit speaking-up and silence	No: unclear whether silence was concerned silence	Yes	Yes
2. Presented a single hazard and ascertained safety concerns	No: poor measure; more than one hazard	No: safety concerns could be increased	Yes
3. Manipulated the perception of risk, not real risk, using deception procedures	No: concerns unclear	Yes	Yes
**THE RELIANCE ON MEMORY AND IMAGINATION**			
4. Recorded behaviours through observation	Yes	Yes	Yes
5. Calibrated safety concerns to elicit speaking-up and safety silence	No: more voice needed	No: still more voice needed	Yes
**The relationship with other variables**			
6. Built a protocol that can manipulate variables of interest	Yes	Yes	Yes
7. Sampled participants using random procedures	Yes	Yes	Yes
8. Minimised alternative mitigations of the hazard	No: some participants did not fully sit	Yes	Yes
9. Provided a clearly specified study protocol	Yes	Yes	Yes

Furthermore, Study 3 revealed that the four types of safety voice behaviours (i.e., concerned voice, concerned silence, unconcerned voice, unconcerned silence) were associated with different levels of fear, felt obligation and care for the research assistant.

## General Discussion

Our results establish a novel experimental paradigm for safety voice. Through an iterative process, three studies addressed nine requirements for a valid safety voice experiment (see Table 1). The final protocol can facilitate behavioural investigations of safety voice, overcome the reliance on memory and recall inherent in report methodologies, and allow for the study of relationships between safety voice and other variables. It is also the first generalisable experimental paradigm for safety voice, enables the investigation of (un)concerned voice and silence, can be used to investigate the effect of safety voice interventions, and through focussing on behaviour, can improve the conceptualisation and operationalisation of safety voice.

### Conceptualising and Operationalising Safety Voice

Through the process of developing the “Walking the plank” paradigm, insights were drawn on conceptualising and operationalising safety voice, the relationship between having and raising safety concerns, the important role of safety silence, and existence of unconcerned voice and silence.

First, studies of safety voice would benefit from operationalising the phenomenon as observable behaviours in response to safety concerns rather than reportable acts. In our studies, it was notable that the presentation of the hazardous footbridge elicited observable safety voice behaviours, and that these often differed from reported safety voice (i.e., about 1 in 5 participants misreported their behaviour, and participants tended to favour misreporting safety voice over silence). This finding reinforces the problems we raised with report methodologies at the outset, and has implications for conclusions from practice-based and academic investigations. Whilst practice-based investigations occur in response to real hazards that elicited safety concerns, these frequently rely on reports of incidents occurred in the past (e.g., interviews, focus groups; Francis, 2013). Furthermore, existing academic studies using behavioural observations (Kolbe et al., 2012, 2014; Barzallo Salazar et al., 2014; Hughes et al., 2014; Sundqvist and Carlsson, 2014; Aubin and King, 2015; Hu et al., 2015; Reime et al., 2016) have not addressed the extent that safety concerns lead to speaking-up (e.g., they assumed that an unsafe procedure causes concerns). Other scholars have merely discussed the behaviourial nature (Hofmann et al., 2003; Jones and Durbridge, 2016), sampled experiences close to the behaviour (Kines et al., 2010) or provided high fidelity simulation training on the behaviour (e.g., Hanson, 2017) without measuring it through behavioural observations. Accordingly, more measures of safety voice should operationalise (or triangulate with) direct observations of behaviours. We showed these behaviours emerged in response to a hazard (i.e., a hazard should be presented, also see below) to prevent physical harm (i.e., safety voice occurs before the hazardous scenario has finished: voice after the scenario may aim to clarify a mismatch between safety information and the results of the scenario).

Second, safety voice is thus rooted in hazard perception: it primarily occurs in response to being presented with a hazardous situation. Yet, the relationship between safety voice and hazards is not straightforward, with hazards emerging through interactions between behaviour and physical contexts, and safety voice occurring when hazard perceptions trigger a safety concern. That is, crucially, whilst the plank of wood became unsafe when the context of its utilisation changed (e.g., from being held up as a mirror, to being used as a low-weight carrying footbridge), our data illustrated that perceiving this as risky only related to safety voice because the risk of breaking concerned participants. Furthermore, we illustrated that (i) an objectively safe plank could be perceived as unsafe before someone walked the footbridge, (ii) not everyone held a safety concern despite safety information regarding the plank's maximum load and perceptions that walking the plank would break it, and (iii) some participants spoke up about safety without reporting feeling concerned. This indicates that safety concerns are based in risk perceptions that are future orientated (i.e., consequences have not yet occurred), uncertain (i.e., there is no direct evidence available to participants regarding consequences of hazards), and subjective (i.e., desirability of physical harm is a personal preference). Thus, safety voice behaviours are not just indicative of the willingness of a participant to raise a safety concern, but reflect more nuanced and subjective judgements rooted in the uncertainty of perceiving hazards, the interplays between actions and objects, and attribution of desirability.

Third, we learned that safety voice behaviours could be codable as a dichotomous variable (i.e., safety voice was observed: yes/no) but were expressed in different utterances which might be better captured in future as a more continuous variable. For example, people raised their safety concern through stating the facts (e.g., “there was a 30 kg weight limit”), exclaiming concern (e.g., “No, don't do that!”), asking for additional information (e.g., “wait, how heavy are you?”), polite statement (e.g., “this should be safe for a kid”), and some participants persisted or physically blocked the research assistant from engaging with the plank. Coding these behaviours in a binary manner enabled statistical analyses (e.g., logistic regressions) and conclusions on the extent that participants raised their safety concerns, yet this variety underscores the need expressed by others to understand voice concepts as conversational acts that can be expressed in different ways (Manning, 2006; Lyndon, 2008; Bashshur and Oc, 2014; Jones and Kelly, 2014; Kulig and Blanchard, 2016). Binary approaches may oversimply otherwise meaningful utterance as silence (Jones and Kelly, 2014), or obscure moderators and drivers of outcomes (Bashshur and Oc, 2014), and crew resource trainings may benefit from understanding the breadth of conversational techniques employed to raise concerns. The Walking the plank paradigm provides a new methodology for exploring this, and particularly enables data collection on variation in utterances to standardised and controlled hazards.

Fourth, we learned that operationalising safety silence behaviours is deeply challenging. Developing strong safety concern measures is important for assessing whether participants withhold concerns, but ascertaining safety concerns is more difficult than observing safety voice behaviours because concerns are intrinsically subjective (as per the above), and cannot be observed directly. Crucially, and echoing the literature on employee voice (Morrison, 2014), for investigating safety voice it is important to ascertain whether participants were concerned. We addressed this through triangulating risk perception and safety concern measures, and alternative operationalisations of safety concerns may be developed for the assessment of safety silence during or before the exposure to the hazard (e.g., physiological measures).

Fifth, through obtaining data on safety concerns we indicated voice and silence for people who were concerned and unconcerned, and the existence of these four types of safety voice merits investigation and conceptualisation. Unconcerned voice may be explained as verbalised sense-making on safety, caution, or a misrepresentation of being concerned, and unconcerned silence may be a misrepresentation or an unawareness of the hazard. In particular, our results suggested that (i) people who withheld their concerns worried more (e.g., about being seen as trouble-maker), cared less about the research assistant, and felt less obligated to raise concerns; (ii) those who spoke-up despite perceiving no safety issues felt less responsible for the research assistant; and (iii) those who were silent and unconcerned simply did not perceive an issue, but would have felt responsible for raising it. Thus, our paradigm enables the development of safety voice into a two-dimensional typology for (un)concerned voice and silence that is rooted in social interaction and sense-making (see Table 7), and this may resemble signal detection typologies (i.e., hit, miss, false positive, false negative; Nesse, 2005).

**Table 7:** Safety voice typology.

	**Safety concern**	**No safety concern**
Voice	Concerned Voice	Unconcerned Voice
Silence	Concerned Silence	Unconcerned Silence

### New Directions for the Investigation of Safety Voice

The literature of safety voice has established a considerable body of findings, and the “Walking the plank” paradigm enables four potential research questions.

#### Can Safety Voice Antecedents Be Replicated?

The paradigm can be used to replicate empirical findings within an experiment setting. We illustrated that safety voice behaviours can be elicited and directly observed, and that self-reports of safety voice were imperfect and biased toward speaking-up. This might raise doubts on report-based evidence regarding the relationship between antecedents and safety voice, and interventions based on these conclusions. For example, self-report and correlation studies indicate that expectations of negative consequences of speaking-up (e.g., Bickhoff et al., 2016) and power hierarchies (e.g., Seiden et al., 2006) can lead to withholding safety concerns. However, whilst power hierarchies have been experimentally manipulated (Barzallo Salazar et al., 2014; Schwappach and Gehring, 2014c; Anicich et al., 2015), simulated (Aubin and King, 2015; Reime et al., 2016), trained on (Hanson, 2017), or discussed as part of single-cases (Liao et al., 2014), to date causality remains unclear because no studies have simultaneously (i) manipulated power hierarchies (e.g., through creating a control condition) and (ii) obtained behavioural data on safety voice (treating reports of the behaviour as empirically different from the behaviour itself). Using the “Walking the plank” paradigm, research can establish whether current findings are upheld in an experimental setting, and establish the causal relationship between safety voice and other variables.

#### What Characterises Safety Voice Behaviours?

We demonstrated safety voice as a behavioural phenomenon, and this opens up new questions for how the behaviour can be characterised. Our experimental paradigm enables the identification of the nature of safety voice behaviours, and especially opens up the investigation of question regarding sense-making, decision-making, physiological mechanisms and linguistic expressions. In particular, although the literature has conceptualised safety voice (i.e., raising concerns held) and silence (i.e., withholding concerns held), we provided the first demonstration and initial conceptualisation of the act of raising safety concerns (and staying silent) when individuals are unconcerned. Through refining the conceptualisation of (un)concerned voice and silence, and testing its predictive power in relationship to safety voice antecedents or outcomes would provide clarity on the nature of safety voice behaviours, and enable targeted interventions.

In addition, we showed that safety voice appears distinct from employee voice. Speaking-up to change expected outcomes (as the verbal behaviour labelled “voice”) is at the core of employee and safety voice, and scope for integration may exist under conceptual overlap (Wilkinson et al., 2019). However, conceptually employee voice includes a broader set of behaviours (i.e., promotive and prohibitive; Liang et al., 2012) than safety voice (i.e., preventing harm is prohibitive in nature), and we found no empirical support that safety voice is a sub-type of employee voice. This may be because, in hazardous situations, safety voice emphasises the prevention of harm based on an assessment of perceived risks that can be ambiguous (e.g., because they are yet to occur, have not been noticed, it is not clear who is responsible, or people are discouraged to raise concerns). This may prompt sensemaking on potential harms (with or without the interlocuter) and felt responsibilities for harm-prevention, and a clearer responsibility and need for sensemaking may lie in warning others than challenging their task-related choices. This underscores the need to consider the content of the raised message through speaking-up (see also: Morrison, 2011), and this extends to the breadth of harmful issues that are raised through safety voice. Devising studies that manipulate the content of safety voice (i.e., judgements on risk, attributions of desirability, types of harm) provides a way to understand voice behaviours.

Third, the direct observation of safety voice behaviours enables the assessment of the decision-making processes regarding whether to raise concerns or not. Decisions to raise safety concerns may be automatic or deliberative, and the optimal way to make decisions for speaking-up about safety remains unexplored. Evidence suggests that people are more inclined to engage in pro-social behaviours under time-pressure (Rand et al., 2014) or in a state of pro-social disinhibition (van den Bos et al., 2009, 2011a,b), and intuitive decisions are frequently implicated (Rand and Epstein, 2014) and effective (Kahneman and Klein, 2009) in preventing harm based on recognising patterns in the situation (e.g., fire fighters recognising that smoke patterns indicate a potentially lethal backdraft). Through manipulating the time-pressure, the paradigm may therefore unearth decision-making mechanisms for safety voice.

A fourth area for conceptualising safety voice behaviours is the association of safety voice with physiological measures. Because the proposed paradigm enables the observation of safety voice *in-situ*, these behaviours can be simultaneously assessed with physiological mechanisms: safety beliefs emerge from embodied experiences (Somerville, 2006) and consequences of silence might manifest physiologically. Scholars may therefore explore the generalisation of safety concerns from physiological mechanisms. Our paradigm lends itself for the inclusion of physiological measures (e.g., skin conductance, heart rate, inhalation, gross movement, vocal amplitude, vocal pitch) and this enables the conceptualisation of the physiological mechanisms underpinning safety voice (e.g., arousal) that can be triangulated to safety concerns measures.

Finally, researchers may utilise the safety voice paradigm to examine the linguistic nature of safety voice. We, in line with others in the literature (e.g., Okuyama et al., 2014), treated safety voice as a binary variable. Yet the manner (e.g., mirroring conversation partners' language, using polite expressions, providing support and explanations, prompts and suggestions; Liu et al., 2016) and intensity (e.g., U. Fischer and Orassanu, 2000) in which people raise safety concerns varies. The “Walking the plank” paradigm provides direct access to safety voice as speaking-up through the systematic observation of conversational processes in response to an observed risk, and novel insights may be drawn through conversation (e.g., on the dialogical patterns; Kendrick, 2017) or speech analysis (e.g., pitch; van Heuven and Boersma, 2001; Kawahara and Morise, 2011). We intend to test and present the linguistic nature of safety voice in future research.

#### Does Personal Relevance of Harm Shape Safety Voice?

We illustrated that safety voice can be observed in laboratory environments, and this enables the investigation of how the personal implication of physical harm shapes safety voice. The behaviour can emerge from individuals who are directly impacted by the consequences of a hazard (e.g., the person walking the plank) or observe others putting themselves in danger, and this may alter results. Different predictions exist for why people prevent harm to others vs. oneself (Crockett et al., 2014), and these emerge from (i) a stronger aversion to one's own pain (i.e., economic exchange hypothesis), (ii) an aversion to conflicting harm (i.e., guilt-aversion hypothesis), and (iii) an equal evaluation of harm to self and others (i.e., empathy perspective). For example, Batson and colleagues found that perceiving someone as needing help increases empathic concern and helping (Batson et al., 2007), and our results suggested that victimhood does not influence safety voice. This opens up questions regarding the relationship between victimhood and safety voice, and whether it is explained by processes such as empathy and perspective taking. The “Walking the plank” paradigm enables the investigation of these questions.

#### Can Concepts for Safety Voice and Harm-Prevention Behaviours Be Integrated?

Safety voice appears conceptually related to obedience to authority and the bystander effect, and the experimental paradigm may be used to investigate conceptual and operational overlap. Milgram's behavioural study of obedience (Milgram, 1963) has been reconceptualised as operationalising an act of defiance (Miller et al., 1995) or resistance (Kaposi, 2017) that closely resembles safety voice (i.e., people repeatedly speak up to resist a harmful order from an authority figure), and resistance may represent a special type of safety voice. Similarly, research on bystander interventions of people in need of assistance (Darley and Latane, 1968; Fischer et al., 2011; Bennett et al., 2014) operationalised harm-prevention behaviours and these have included non-verbal (e.g., walking to another room to intervene in sexual harrasment; P. Fischer et al., 2006) and verbal actions (van den Bos et al., 2009). However, the extent of overlap between these harm-prevention behaviours (e.g., raising a concern to a senior figure; Milgram, 1963) and triggers (e.g., the necessity of noticing and evaluating situations as dangerous; Latane and Darley, 1968) remains unaddressed. Evaluating overlap and integrating conceptualisations would provide an interesting research agenda, especially because, to our awareness, we are the first to indicate that unconcerned participants can step up to prevent harm, and the notion of “unconcerned voice” would provide a novel take on long-established paradigms (e.g., disobedient participants might have objected about electric shocks on principle, not due to safety concerns).

### Limitations

Three limitations of the experimental paradigm must be stated. That is, first, the paradigm cannot establish whether safety voice prevents physical harm: the paradigm presents a controlled hazard (i.e., the safety of its actual outcome is assured), and assumes that if the hazard were real physical harm would have been prevented. This is an important limitation that emerges from an ethical paradox: safety voice research aims to design safety interventions, but experiments cannot put participants in harm's way (e.g., actually breaking the plank, violence to participants, etc.). We illustrated how this can be addressed through manipulating risk perceptions that lead to safety concerns.

Second, the responses to the wrap-up questionnaire scales may be interpreted differently by those who raised a concern or remained silent, and may be understood as rationalising their behaviour (P. Fischer et al., 2006). For example, through obtaining safety concerns *post-hoc*, results may be interpreted as self-perception (Bem, 1967): people that remained silent were less likely to say they were concerned. This challenge is common for experiments that use a cover story to introduce a hazard, and we agree with Fischer and colleagues that the presentation of the questionnaire after the behaviour is the optimal procedure to collect additional data “without risking the credibility of [the] experimental design. Asking about these variables right after the danger manipulation and before measuring the dependent variable would have caused suspicion and unmasked our cover story” (p. 272). This underscores the need for direct observations of safety voice, and the development of direct measures of its antecedents (e.g., physiological measures of safety concerns).

Third, the external validity of experiments is debated (Gigerenzer, 1984; Jiménez-Buedo and Miller, 2010) and because conclusions may not generalise (e.g., to the Intensive Care Unit, or mountaineering) the paradigm may thus not provide insights for unique environments. Yet, safety voice behaviours are highly contextual (i.e., they are shaped by antecedents and hazards), and, to enable conclusions on safety voice mechanisms, this calls for strict control over contextual variables through standardised assessments. That is, conclusions with high internal validity are near-impossible to draw in fast-paced environments with inconsistent presentation of antecedents and hazards, and mechanisms can only be established using highly standardised measures or scenarios presented across participants. Providing high internal validity, the proposed paradigm can isolate speaking-up in a standardised scenario and generalise with more certainty to contexts that have (manageable) characteristics tested through the paradigm, and external validity can be established through benchmark findings against other contexts.

## Conclusion

Safety voice behaviours can be observed in laboratory experiments (and safety silence through assessing safety concerns). This is important because current safety voice methodologies have shortfalls, and experimental paradigms, despite their own limitations, are needed to address the behavioural nature of safety voice, reliance on memory and recall, and relationship between safety voice and other variables. We presented the first experimental paradigm for investigating safety voice (the Walking the plank paradigm) that can address the requirements for safety voice experiments, and we illustrated how these can be evaluated. Through investigating safety voice experimentally, insight was provided on the importance of considering risk perception when interpreting behaviour, leading to a new two-dimensional typology for analysing safety voice behaviours. Our presentation of the paradigm adds to the debate on the need for appropriate methodologies for investigating harm prevention behaviours. The literature on safety voice has generated considerable insight into why people raise safety concerns, and the development of experimental methodologies advances the field: fostering the development of behavioural conceptualisations, new directions for research, and stronger interventions for the prevention of physical harm. People speaking-up about safety has saved countless lives, and experimentally examining the causes and nature of this behaviour has the potential to increase the prevalence and effectiveness for people to create safety through speaking-up.

## Data Availability

The datasets generated for this study are available on request to the corresponding author.

## Author Contributions

MN was responsible for designing and conceptualising the study, data collection, formal data analysis and interpretation, and manuscript preparation (80%). TR and AG contributed to the conceptualization of the study, refining the study design, interpreting results, and reviewing and editing the manuscript (20%).

### Conflict of Interest Statement

The authors declare that the research was conducted in the absence of any commercial or financial relationships that could be construed as a potential conflict of interest.

## Footnotes

^1^An online pilot study (*n* = 88), that asked participants to rate a video of the scenario, suggested that participants perceived the hazard equally unsafe dependent on whether they or someone else would be the victim, *F*_(88, 1)_ = 0.03, *p* = 0.86, η^2^ = 0.00.

^2^The manipulation of one experimental variable was explored (i.e., high and low expectations of negative consequences of voice), but this is not discussed here because the condition was randomised and produced no significant main-effect.

^3^A lab-based pilot study (*n* = 38) demonstrated that the mere presentation of the message regarding the maximum load of the plank was sufficient to reproduce safety voice behaviours, *OR* = 17.00, *Wald*(1) = 6.38, *p* = 0.01.

^4^The manipulation of two experimental variables was explored (i.e., perceived hazard awareness of the research assistant; perspective-taking), but these are not discussed here because the conditions were randomised and produced no significant main-effect.

^5^An online pilot study (*n* = 57), that asked participants to rate a video of the scenario, revealed that the act of walking the plank was considered unsafe and likely to lead to undesirable and preventable physical harm by 76% of participants, *t*_(56)_ = 7.28, *p* < 0.001.

^6^A manual is made available as Supplementary Material.

## References

[B1] Aarts H. Custers R. Marien H. (2008). Preparing and motivating behavior outside of awareness. Science 319, 1639–1639. 10.1126/science.1150432 18356517

[B2] Anicich E. M. Swaab R. I. Galinsky A. D. (2015). Hierarchical cultural values predict success and mortality in high-stakes teams. Proc. Natl. Acad Sci. U.S.A. 112, 1338–1343. 10.1073/pnas.1408800112 25605883 PMC4321300

[B3] Aubin D. King S. (2015). Developing a culture of safety: exploring students? perceptions of errors in an interprofessional setting. J. Interprof. Care 29, 646–648. 10.3109/13561820.2015.1045060 26652639

[B4] Bagozzi R. P. Yi Y. Phillips L. W. (1991). Assessing construct validity in organizational research. Admin. Sci. Q. 36, 421–458. Retrieved from http://www.jstor.org/stable/2393203 (accessed March 20, 2019).

[B5] Bartlett F. C. (1932). Remembering: A Study in Experimental and Social Psychology. (Cambridge: Social Psychology), 1–11.

[B6] Barzallo Salazar M. J. Minkoff H. Bayya J. Gillett B. Onoriode H. Weedon J.. (2014). Influence of surgeon behavior on trainee willingness to speak up: a randomized controlled trial. J. Am. Coll. Surg. 219, 1001–1007. 10.1016/j.jamcollsurg.2014.07.933 25256368

[B7] Bashshur M. R. Oc B. (2014). When voice matters: a multilevel review of the impact of voice in organizations. J. Manage. 41, 1530–1554. 10.1177/0149206314558302

[B8] Batson C. D. Eklund J. H. Chermok V. L. Hoyt J. L. Ortiz B. G. (2007). An additional antecedent of empathic concern: valuing the welfare of the person in need. J. Pers. Soc. Psychol. 93, 65–74. 10.1037/0022-3514.93.1.65 17605589

[B9] Baumeister R. F. (1982). A self-presentational view of social phenomena. Psychol. Bull. 91, 3–26. 10.1037/0033-2909.91.1.3

[B10] Bem D. J. (1967). Self-perception: an alternative interpretation of cognitive dissonance phenomena. Psychol. Rev. 74, 183–200. 10.1037/h0024835 5342882

[B11] Bennett S. Banyard V. L. Garnhart L. (2014). To act or not to act, that is the question? Barriers and facilitators of bystander intervention. J. Interpers. Violence 29, 476–496. 10.1177/0886260513505210 24097909

[B12] Bickhoff L. Levett-jones T. Sinclair P. M. (2016). Nurse education today rocking the boat - nursing students' stories of moral courage: a qualitative descriptive study. Nurse Educ. Today 42, 35–40. 10.1016/j.nedt.2016.03.030 27237350

[B13] Bienefeld N. Grote G. (2012). Silence that may kill: when aircrew members don't speak up and why. Aviat. Psychol. Appl. Hum. Fact. 2, 1–10. 10.1027/2192-0923/a000021

[B14] Blanco M. Clarke J. R. Martindell D. Denise M. (2009). Wrong site surgery. AORN J. 90, 814–816. 10.1016/j.aorn.2009.07.010 19664413

[B15] Blass T. (1999). The milgram paradigm after 35 years: some things we now know about obedience to authority1. J. Appl. Soc. Psychol. 29, 955–978. 10.1111/j.1559-1816.1999.tb00134.x

[B16] Brodin T. Klaminder J. Fick J. Fahlman J. Jonsson M. Hellström G. (2016). Drug-induced behavioral changes: using laboratory observations to predict field observations. Front. Environ. Sci. 4, 1–9. 10.3389/fenvs.2016.00081

[B17] Carver C. S. White T. L. (1994). Behavioral inhibition, behavioral activation, and affective responses to impending reward and punishment: The BIS/BAS Scales. J. Pers. Soc. Psychol. 67, 319–333. 10.1037/0022-3514.67.2.319

[B18] Cocklin J. T. (2004). Swissair 111 human factors: checklist and cockpit communications. J. Air Transp. 9 Retrieved from https://search.proquest.com/docview/232853869?accountid=9630 (accessed March 20, 2019).

[B19] Cox D. R. Oakes D. (1984). Analysis of Survival Data. London: Chapman & Hall.

[B20] Crockett M. J. Kurth-Nelson Z. Siegel J. Z. Dayan P. Dolan R. J. (2014). Harm to others outweighs harm to self in moral decision making. Proc. Natl Acad. Sci. 111, 17320–17325. 10.1073/pnas.1408988111 25404350 PMC4260587

[B21] Custers R. Aarts H. (2010). The unconscious will: how the pursuit of goals operates outside of conscious awareness. Science 329, 47–50. 10.1126/science.1188595 20595607

[B22] Darley J. M. Latane B. (1968). Bystander intervention in emergencies: diffusion of responsibility. J. Pers. Soc. Psychol. 8(4 Pt 1), 377–383. 10.1037/h0025589 5645600

[B23] Del Boca F. K. Noll J. A. (2000). Truth or consequences: the validity of self-report data in health services research on addictions. Addiction 95, 347–360. 10.1046/j.1360-0443.95.11s3.5.x 11132362

[B24] Delisle M. Grymonpre R. Whitley R. Wirtzfeld D. (2016). Crucial conversations: an interprofessional learning opportunity for senior healthcare students. J. Interprof. Care 30, 777–786. 10.1080/13561820.2016.1215971 27715347

[B25] Earp B. D. Trafimow D. (2015). Replication, falsification, and the crisis of confidence in social psychology. Front. Psychol. 6:621. 10.3389/fpsyg.2015.00621 26042061 PMC4436798

[B26] Fischer P. Greitemeyer T. Pollozek F. Frey D. (2006). The unresponsive bystander: are bystanders more responsive in dangerous emergencies? Eur. J. Soc. Psychol. 36, 267–278. 10.1002/ejsp.297

[B27] Fischer P. Krueger J. I. Greitemeyer T. Vogrincic C. Kastenmüller A. Frey D.. (2011). The bystander-effect: a meta-analytic review on bystander intervention in dangerous and non-dangerous emergencies. Psychol. Bull. 137, 517–537. 10.1037/a0023304 21534650

[B28] Fischer U. Orassanu J. (2000). Error-challenging strategies: their role in preventing and correcting errors, in Proceedings of the Human Factors and Ergonomics Society Annual Meeting (Los Angeles, CA: SAGE Publications), 30–33.

[B29] Francis R. (2013). Report of the Mid Staffordshire NHS Foundation Trust Public Inquiry Executive Summary. Report of the Mid Staffordshire NHS Foundation Trust Public Inquiry. Executive Report.

[B30] Francis R. (2015). Freedom to Speak Up: An Independet Review into Creating an Open and Honest Reporting Culture in the NHS. Retrieved from https://freedomtospeakup.org.uk/wp-content/uploads/2014/07/F2SU_web.pdf (accessed March 20, 2019).

[B31] Gergen K. J. (1973). Social psychology as history. J. Pers. Soc. Psychol. 26, 309–320. 10.1037/h0034436

[B32] Gigerenzer G. (1984). External validity of laboratory experiments: the frequency-validity relationship. Am. J. Psychol. 97:185 10.2307/1422594

[B33] Gkorezis P. Panagiotou M. Theodorou M. (2016). Workplace ostracism and employee silence in nursing: the mediating role of organizational identification. J. Adv. Nurs. 72, 2381–2388. 10.1111/jan.12992 27113971

[B34] Goodwin C. J. (2008). Research in Psychology Methods and Design, 5th Edn. Hoboken, NJ: John Wiley & Sons.

[B35] Habyarimana J. Jack W. (2011). Heckle and chide: results of a randomized road safety intervention in Kenya. J. Public Econ. 95, 1438–1446. 10.1016/j.jpubeco.2011.06.008

[B36] Hanson D. (2017). Reducing central line-associated bloodstream infection rates in the context of a caring-healing environment. J. Infus. Nurs. 40, 101–110. 10.1097/NAN.0000000000000212 28248810

[B37] Hodges S. (2018). The Effect of Relationship Strength on Safety Voicing. University of Canterbury. Retrieved from http://hdl.handle.net/10092/15072 (accessed March 20, 2019).

[B38] Hofmann D. A. Morgeson F. P. Gerras S. J. (2003). Climate as a moderator of the relationship between leader-member exchange and content specific citizenship: safety climate as an exemplar. J. Appl. Psychol. 88, 170–178. 10.1037/0021-9010.88.1.170 12675404

[B39] Hu Y-Y. Parker S. H. Lipsitz S. R. Arriaga A. F. Peyre S. E. Corso K. A.. (2015). Surgeons' leadership styles and team behavior in the operating room. J. Am. Coll. Surg. 222, 41–51. 10.1016/j.jamcollsurg.2015.09.013 26481409 PMC4769879

[B40] Hughes K. M. Benenson R. S. Krichten A. E. Clancy K. D. Ryan J. P. Hammond C. (2014). A crew resource management program tailored to trauma resuscitation improves team behavior and communication. J. Am. Coll. Surg. 219, 545–551. 10.1016/j.jamcollsurg.2014.03.049 25026871

[B41] Jiménez-Buedo M. Miller L. M. (2010). Why a trade-off? the relationship between the external and internal validity of experiments. Int. J. Theory 25, 301–321. Retrieved from http://www.ehu.es/ojs/index.php/THEORIA/article/view/779/700 (accessed March 20, 2019).

[B42] Johnson H. L. Kimsey D. (2012). Patient safety: break the silence. AORN J. 95, 591–601. 10.1016/j.aorn.2012.03.002 22541770

[B43] Jones A. Kelly D. (2014). Deafening silence? Time to reconsider whether organisations are silent or deaf when things go wrong. BMJ Qual. Saf. 23, 1–5. 10.1136/bmjqs-2013-002718 25015116

[B44] Jones C. Durbridge M. (2016). Culture, silence and voice: the implications for patient safety in the operating theatre. J. Perioper. Pract. 26, 281–284. 10.1177/175045891602601204 29328767

[B45] Kahneman D. Klein G. (2009). Conditions for intuitive expertise: a failure to disagree. Am. Psychol. 64, 515–526. 10.1037/a0016755 19739881

[B46] Kaposi D. (2017). The resistance experiments: morality, authority and obedience in Stanley Milgram's account. J. Theory Soc. Behav. 47, 382–401. 10.1111/jtsb.12137

[B47] Kawahara H. Morise M. (2011). Technical foundations of TANDEM-STRAIGHT, a speech analysis, modification and synthesis framework. Sadhana 36, 713–727. 10.1007/s12046-011-0043-3

[B48] Kendrick K. H. (2017). Using conversation analysis in the lab. Res. Lang. Soc. Interact. 50, 1–11. 10.1080/08351813.2017.1267911

[B49] Kines P. Andersen L. P. S. S. Spangenberg S. Mikkelsen K. L. Dyreborg J. Zohar D. (2010). Improving construction site safety through leader-based verbal safety communication. J. Safety Res. 41, 399–406. 10.1016/j.jsr.2010.06.005 21059457

[B50] Kolbe M. Burtscher M. J. Wacker J. Grande B. Nohynkova R. Manser T.. (2012). Speaking up is related to better team performance in simulated anesthesia inductions: an observational study. Anesth. Analg. 115, 1099–1108. 10.1213/ANE.0b013e318269cd32 23011565

[B51] Kolbe M. Grote G. Waller M. J. Wacker J. Grande B. Burtscher M. J.. (2014). Monitoring and talking to the room: autochthonous coordination patterns in team interaction and performance. J. Appl. Psychol. 99, 1254–1267. 10.1037/a0037877 25222522

[B52] Kulig A. W. Blanchard R. D. (2016). Use of cognitive simulation during anesthesiology resident applicant interviews to assess higher-order thinking. J. Grad. Med. Educ. 8, 417–421. 10.4300/JGME-D-15-00367.1 27413447 PMC4936862

[B53] Lappi O. Rinkkala P. Pekkanen J. (2017). Systematic observation of an expert driver's gaze strategy-an on-road case study. Front. Psychol. 8:620. 10.3389/fpsyg.2017.00620 28496422 PMC5406466

[B54] Latane B. Darley J. M. (1968). Group inhibition of bystander intervention in emergencies. J. Pers. Soc. Psychol. 10, 215–221. 10.1037/h0026570 5704479

[B55] Liang J. Farh C. I. C. Farh J-L. (2012). Psychological antecedents of promotive and prohibitive voice: a two-wave examination. Acad. Manage. J. 55, 71–92. 10.5465/amj.2010.0176

[B56] Liao J. M. Thomas E. J. Bell S. K. (2014). Speaking up about the dangers of the hidden curriculum. Health Aff. 33, 168–171. 10.1377/hlthaff.2013.1073 24395948

[B57] Liu W. Gerdtz M. Manias E. (2016). Creating opportunities for interdisciplinary collaboration and patient-centred care: how nurses, doctors, pharmacists and patients use communication strategies when managing medications in an acute hospital setting. J. Clin. Nurs. 25, 2943–2957. 10.1111/jocn.13360 27550739

[B58] Lyndon A. (2008). Social and environmental conditions creating fluctuating agency for safety in two urban academic birth centers. J. Obstet. Gynecol. Neonatal Nurs. 37, 13–23. 10.1111/j.1552-6909.2007.00204.x 18226153

[B59] Manapragada A. Bruk-lee V. (2016). Staying silent about safety issues: conceptualizing and measuring safety silence motives. Accid. Anal. Prevent. 91, 144–156. 10.1016/j.aap.2016.02.014 26974031

[B60] Manias E. (2015). Communication relating to family members' involvement and understandings about patients' medication management in hospital. Health Expect. 18, 850–866. 10.1111/hex.12057 23405906 PMC5060807

[B61] Manning M. L. (2006). Patient safety improving clinical communication through structured conversation. Nursing 24, 268–272. 17131620

[B62] March J. G. Olsen J. P. (1975). The uncertainty of the past: organizational learning under ambiguity. Eur. J. Polit. Res. 3, 147–171. 10.1111/j.1475-6765.1975.tb00521.x

[B63] Marshall K. G. (1996). Prevention. how much harm? How much benefit? 3. physical, psychological and social harm. CMAJ. 155, 169–176. 8800074 PMC1487962

[B64] Mastrofski S. D. Parks R. B. McCluskey J. D. (2010). Systematic social observation in criminology, in Handbook of Quantitative Criminology, eds Piquero A. R. Weisburd D. (New York, NY: Springer), 225–247.

[B65] Mastrofski S. D. Parks R. B. Reiss A. J. Jr. Worden R. E. DeJong C. Snipes J. B. (1998). Systematic Observation of Public Police: Applying Field Research Methods to Policy Issues. Washington, DC: National Institute of Justice Retreived from https://www.ncjrs.gov/pdffiles/172859.pdf (accessed March 20, 2019).

[B66] McBee M. (2010). Modeling outcomes with floor or ceiling effects: an introduction to the tobit model. Gifted Child Q. 54, 314–320. 10.1177/0016986210379095

[B67] Milgram S. (1963). Behavioral study of obedience. J. Abnorm. Psychol. 67, 371–378. 14049516 10.1037/h0040525

[B68] Milgram S. (1974). Obedience to Authority: An Experimental View. New York, NY: Harper & Row.

[B69] Miller A. G. Collins B. E. Brief D. E. (1995). Perspectives on obedience to authority: the legacy of the milgram experiments. J. Soc. Issues 51, 1–19. 10.1111/j.1540-4560.1995.tb01331.x

[B70] Moorhead G. Ference R. Neck C. P. (1991). Group decision fiascoes continue: space shuttle challenger and a revised groupthink framework. Hum. Relat. 44, 539–550. 10.1177/001872679104400601

[B71] Morrison E. W. (2011). Employee voice behavior: integration and directions for future research. Acad. Manag. Ann. 5, 373–412. 10.5465/19416520.2011.574506

[B72] Morrison E. W. (2014). Employee voice and silence. Annu. Rev. Organ. Psychol. Organ. Behav. 1, 173–197. 10.1146/annurev-orgpsych-031413-091328

[B73] Morrison E. W. See K. E. Pan C. (2015). An approach-inhibition model of employee silence: the joint effects of personal sense of power and target openness. Pers. Psychol. 68, 547–580. 10.1111/peps.12087

[B74] Morrow K. J. Gustavson A. M. Jones J. (2016). Speaking up behaviours (safety voices) of healthcare workers: a metasynthesis of qualitative research studies. Int. J. Nurs. Stud. 64, 42–51. 10.1016/j.ijnurstu.2016.09.014 27684321

[B75] Mulhall A. (2003). Methodological issues in nursing research in the field: notes on observation in qualitative research. J. Adv. Nurs. 3, 306–313. 10.1046/j.1365-2648.2003.02514.x 12581118

[B76] Murphy E. Dingwall R. (2007). Informed consent, anticipatory regulation and ethnographic practice. Soc. Sci. Med. 65, 2223–2234. 10.1016/j.socscimed.2007.08.008 17888553

[B77] Nembhard I. M. Labao I. Savage S. (2015). Breaking the silence. Health Care Manage. Rev. 40, 225–236. 10.1097/HMR.0000000000000028 24901299

[B78] Nesse R. M. (2005). Natural selection and the regulation of defenses. A signal detection analysis of the smoke detector principle. Evol. Hum. Behav. 26, 88–105. 10.1016/j.evolhumbehav.2004.08.002

[B79] Nichols A. L. Maner J. K. (2008). The good-subject effect: investigating participant demand characteristics. J. Gen. Psychol. 135, 151–166. 10.3200/GENP.135.2.151-166 18507315

[B80] Okuyama A. Wagner C. Bijnen B. (2014). Speaking up for patient safety by hospital-based health care professionals: a literature review. BMC Health Serv. Res. 14:61. 10.1186/1472-6963-14-61 24507747 PMC4016383

[B81] Pérez-Tejera F. Valera S. Anguera M. T. (2018). Using systematic observation and polar coordinates analysis to assess gender-based differences in park use in Barcelona. Front. Psychol. 9:2299. 10.3389/fpsyg.2018.02299 30542307 PMC6277760

[B82] Podsakoff P. M. Organ D. W. (1986). Self-reports in organizational research: problems and prospects. J. Manage. 12, 531–544. 10.1177/014920638601200408

[B83] Rand D. G. Epstein Z. G. (2014). Risking your life without a second thought: intuitive decision-making and extreme altruism. PLoS ONE 9:e109687. 10.1371/journal.pone.0109687 25333876 PMC4198114

[B84] Rand D. G. Peysakhovich A. Kraft-Todd G. T. Newman G. E. Wurzbacher O. Nowak M. A.. (2014). Social heuristics shape intuitive cooperation. Nat. Commun. 5:3677. 10.1038/ncomms4677 24751464

[B85] Reader T. W. O'Connor P. (2014). The deepwater horizon explosion: non-technical skills, safety culture, and system complexity. J. Risk Res. 17, 405–424. 10.1080/13669877.2013.815652

[B86] Reime M. H. Johnsgaard T. Kvam F. I. Aarflot M. Breivik M. Engeberg J. M.. (2016). Simulated settings; powerful arenas for learning patient safety practices and facilitating transference to clinical practice. A mixed method study. Nurse Educ. Pract. 21, 75–82. 10.1016/j.nepr.2016.10.003 27769018

[B87] Reiss A. J. (1971). Systematic observation of natural social phenomena. Sociol. Methodol. 3, 3–33. 10.2307/270816

[B88] Rogers W. P. (1986). Report of the Presidential Commission on the Space Shuttle Challenger Accident. Washington, DC Retrieved from https://spaceflight.nasa.gov/outreach/SignificantIncidents/assets/rogers_commission_report.pdf (accessed March 20, 2019).

[B89] Rydenfält C. Johansson G. Odenrick P. Åkerman K. Larsson P. A. (2015). Distributed leadership in the operating room: a naturalistic observation study. Cogn. Technol. Work 17, 451–460. 10.1007/s10111-014-0316-9

[B90] Schwabe L. Wolf O. T. (2009). The context counts: congruent learning and testing environments prevent memory retrieval impairment following stress. Cogn. Affect. Behav. Neurosci. 9, 229–236. 10.3758/CABN.9.3.229 19679758

[B91] Schwappach D. L. B. Gehring K. (2014a). Frequency of and predictors for withholding patient safety concerns among oncology staff: a survey study. Eur. J. Cancer Care 24, 395–403. 10.1111/ecc.12255 25287114

[B92] Schwappach D. L. B. Gehring K. (2014b). “Saying it without words”: a qualitative study of oncology staff's experiences with speaking up about safety concerns. BMJ Open 4:e004740 10.1136/bmjopen-2013-004740 PMC4025461 24838725

[B93] Schwappach D. L. B. Gehring K. (2014c). Silence that can be dangerous: a vignette study to assess healthcare professionals' likelihood of speaking up about safety concerns. PLoS ONE 9:e104720. 10.1371/journal.pone.0104720 25116338 PMC4130576

[B94] Schwappach D. L. B. Gehring K. (2014d). Trade-offs between voice and silence: a qualitative exploration of oncology staff's decisions to speak up about safety concerns. BMC Health Serv. Res. 14:303. 10.1186/1472-6963-14-303 25017121 PMC4105519

[B95] Schwarz N. Bless H. Strack F. Klumpp G. (1991). Ease of retrieval as information: another look at the availability heuristic. J. Pers. Soc. Psychol. 61, 195–202. 10.1037/0022-3514.61.2.195

[B96] Seiden S. C. Galvan C. Lamm R. (2006). Enhancing patient safety. Qual. Saf. Health Care 15, 272–277. 10.1136/qshc.2006.018044 16885252 PMC2564025

[B97] Sheeran P. (2002). Intention — behavior relations: a conceptual and empirical review. Eur. Rev. Soc. Psychol. 12, 1–36. 10.1080/14792772143000003

[B98] Somerville M. (2006). Subjected bodies, or embodied subjects: subjectivity and learning safety at work, in Work, Subjectivity and Learning (1994), 37–52. Retrieved from http://link.springer.com/chapter/10.1007/1-4020-5360-6_3 (accessed March 20, 2019).

[B99] Stoffregen T. A. Bardy B. G. Smart L. J. Pagulayan R. (2003). On the nature and evaluation of fidelity in virtual environments, in Virtual and Adaptive Environments: Applications, Implications, and Human Performance Issues, eds Hettinger L. J. Haas M. (London: Lawrence Erlbauw Associates), 111–128.

[B100] Sundqvist A. S. Carlsson A. A. (2014). Holding the patient's life in my hands: Swedish registered nurse anaesthetists' perspective of advocacy. Scand. J. Caring Sci. 28, 281–288. 10.1111/scs.12057 23713584

[B101] Tamuz M. Thomas E. J. (2006). Classifying and interpreting threats to patient safety in hospitals: insights from aviation. J. Organ. Behav. 27, 919–940. 10.1002/job.419

[B102] Tarnow E. (1999). Self-destructive obedience in the airplane cockpit and the concept of obedience optimization, in Obedience to Authority, ed Blass T. (New York, NY: Psychology Press), 125–138.

[B103] Tucker S. Chmiel N. Turner N. Sandy Hershcovis M. Stride C. B. (2008). Perceived organizational support for safety and employee safety voice: the mediating role of coworker support for safety. J. Occup. Health Psychol. 13, 319–330. 10.1037/1076-8998.13.4.319 18837627

[B104] van de Mortel T. (2008). Faking it: social desirability response bias in self- report research. Aust. J. Adv. Nurs. 25, 40–48.

[B105] van den Bos K. Muller P. A. Damen T. (2011a). A behavioral disinhibition hypothesis of interventions in moral dilemmas. Emotion Rev. 3, 281–283. 10.1177/1754073911402369

[B106] van den Bos K. Müller P. A. van Bussel A. A. L. (2009). Helping to overcome intervention inertia in bystander's dilemmas: behavioral disinhibition can improve the greater good. J. Exp. Soc. Psychol. 45, 873–878. 10.1016/j.jesp.2009.03.014

[B107] van den Bos K. Van Lange P. A. M. Lind E. A. Venhoeven L. A Beudeker D. A Cramwinckel F. M.. (2011b). On the benign qualities of behavioral disinhibition: because of the prosocial nature of people, behavioral disinhibition can weaken pleasure with getting more than you deserve. J. Pers. Soc. Psychol. 101, 791–811. 10.1037/a0023556 21574725

[B108] Van Dyne L. Ang S. Botero I. C. (2003). Conceptualizing employee silence and employee voice as multidimensional constructs. J. Manage. Stud. 40, 1359–1392. 10.1111/1467-6486.00384

[B109] van Heuven V. Boersma P. (2001). Speak and unSpeak with PRAAT. Glot Int. 5, 341–347.

[B110] van Schagen I. Sagberg F. (2012). The potential benefits of naturalistic driving for road safety research: theoretical and empirical considerations and challenges for the future. Procedia Soc. Behav. Sci. 48, 692–701. 10.1016/j.sbspro.2012.06.1047

[B111] Waller M. J. Kaplan S. A. (2016). Systematic behavioral observation for emergent team phenomena. Organ. Res. Methods 21, 500–515. 10.1177/1094428116647785

[B112] Weathington B. L. Cunningham C. J. L. Pittenger D. J. (2010). Research Methods for the Behavioral and Social Sciences. (Hoboken, NJ: John Wiley & Sons).

[B113] Wei X. Zhang Z-X. Chen X-P. (2015). I will speak up if my voice is socially desirable: a moderated mediating process of promotive versus prohibitive voice. J. Appl. Psychol. 100, 1641–1652. 10.1037/a0039046 25844927

[B114] Wilkinson A. Barry M. Morrison E. (2019). Toward an integration of research on employee voice. Hum. Resour. Manage. Rev. 3:1 10.1016/j.hrmr.2018.12.001

