# Supplementary material for: Walking the Plank: An Experimental Paradigm to Investigate Safety Voice
Source: Front Psychol. 2019 Apr 2;10:668. doi: 10.3389/fpsyg.2019.00668 (PMC6454216; doi:10.3389/fpsyg.2019.00668)
Supplement: Supplementary file 1 [file Data_Sheet_1.PDF]

WALKING THE PLANK

A manual for the experimental investigation of safety voice

Mark C Noort

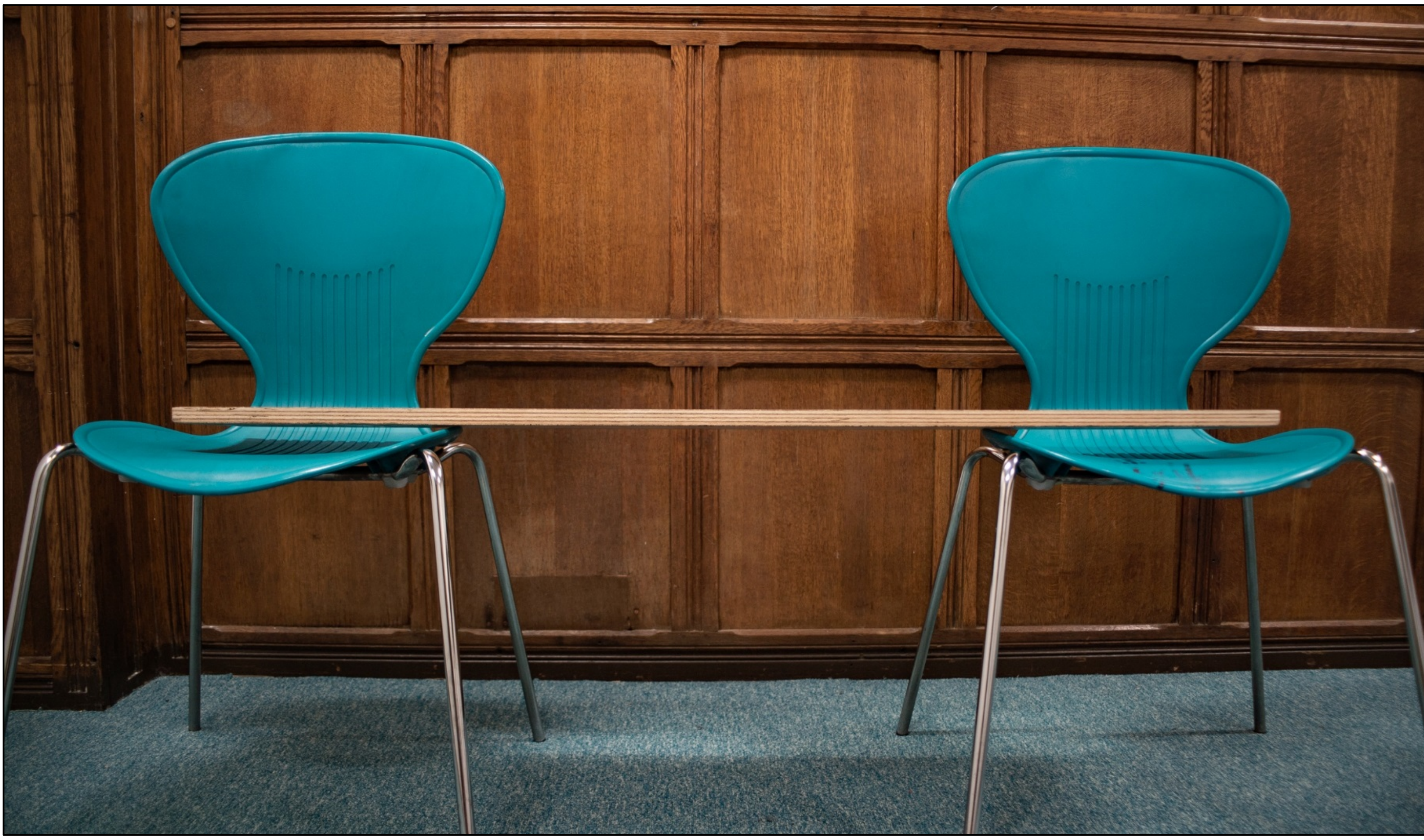

© Mark C Noort, 2019

✉ M.C.Noort@lse.ac.uk / Mark.Noort@outlook.com

INTRODUCTION

'Safety voice' is the act of speaking-up about safety issues. It is defined as "explicit communication that is 1) discretionary, 2) aimed at improving a perceived unsafe situation, and 3) addressed to others of equal or senior status." (Noort, Reader & Gillespie, forthcoming), and through speaking up about safety, hazards can be identified and mitigated.

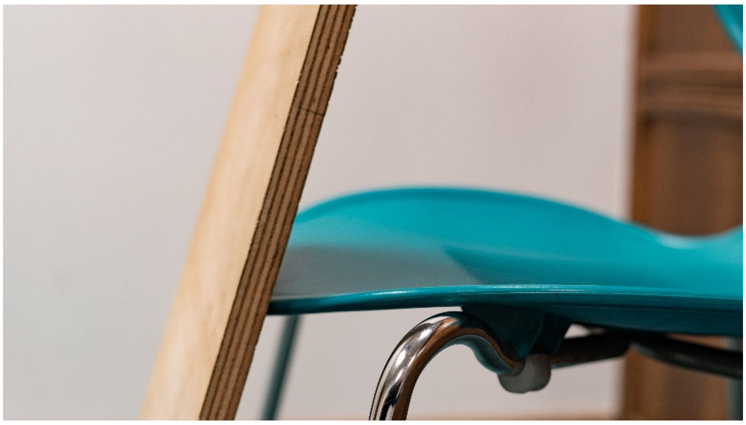

This manual outlines the protocol for the investigation of safety voice through using the Walking the Plank paradigm. Five stages are required to enable the direct observation of safety voice behaviours, and these involve i) participant welcome and informed consent, ii) creativity task, iii) demonstration and evaluation of creative ideas, iv) wrap-up questionnaire, and v) debrief. To enable interpretation and amendments to the protocol, and successful execution of the protocol, we have provided detailed information for each stage of the Walking the Plank paradigm, a checklist for materials and key decisions, appendices to illustrate study materials, illustrative pictures.

The Walking the Plank paradigm makes accessible safety voice behaviours, and you are encouraged to tailor procedures for the purpose of your research. Researching safety voice is fascinating, and we trust that future research will find this paradigm useful for identifying novel concepts for speaking-up about safety, and new ways for people to create safety.

Mark C. Noort

And on behalf of:
Dr Tom W. Reader,
Dr Alex Gillespie.

Contents

INTRODUCTION **3**

WALKING THE PLANK: AN OVERVIEW **5**

General behaviour of research assistants 5
Laboratory environment 6
Materials 6

PREPARATION **7**

Reception Area 7
Observation room 7
Experiment room 7

CHECKLIST **8**

PROTOCOL STEPS **9**

Stage 1: Participant Welcome 9
Stage 2: 'Creativity' Task 10
Stage 3: Demonstration and evaluation of Creative ideas 11
Stage 4: Wrap-up Questionnaire 12
Stage 5: Debrief 13

REFERENCES **14**

APPENDIX A: BRIEF FORM AND INFORMED CONSENT (PAPER VERSION) **15**

APPENDIX B: DEBRIEF FORM (PAPER VERSION) **17**

APPENDIX C: THE CREATIVITY-FEASIBILITY FORM (PAPER VERSION) **18**

WALKING THE PLANK: AN OVERVIEW

The Walking the plank paradigm consists of 3 core stages: i) a creativity task (to introduce the limits of the plank), ii) demonstration and evaluation of creative ideas (to introduce the hazard and observe safety voice/silence), and iii) wrap-up questionnaire (to ascertain safety concerns and study variables of interest). These stages are flanked by a participant welcome (to register participants and obtain informed consent) and debrief (to clarify the true study intent and ensure participants leave in the same psychological state they came in with). The study takes ±30-minutes per participant to complete.

To enable these stages, the protocol puts requirements on the general behaviour of research assistants, the laboratory setting and materials. The checklist provided at the end of this manual summarises these requirements. The pictures illustrate the environment and materials used to develop the paradigm.

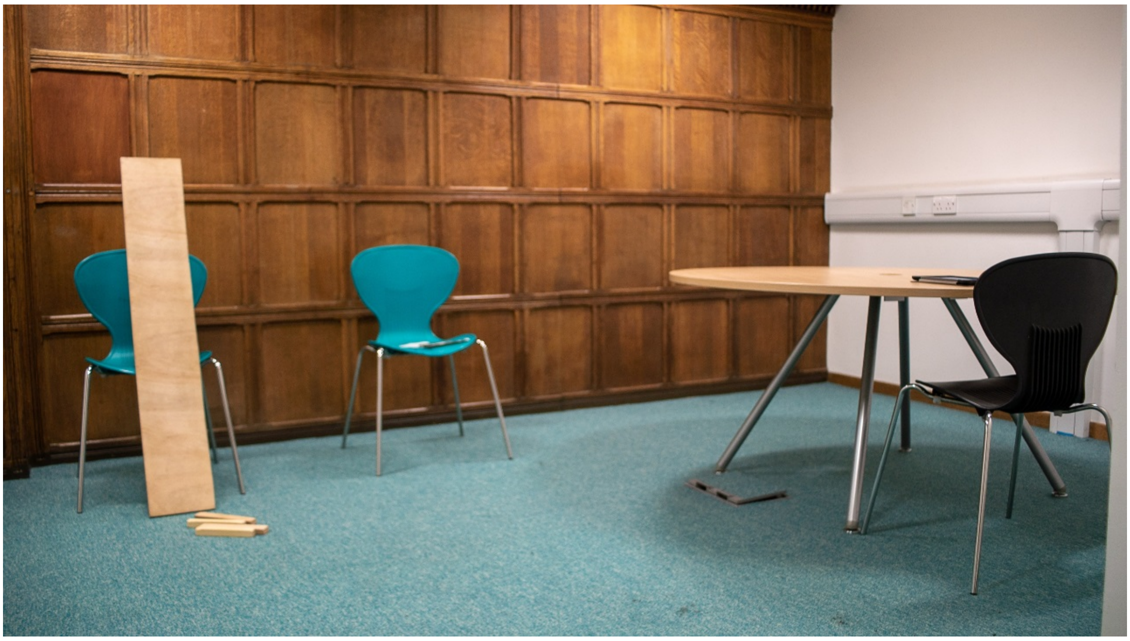

Experiment room with starting position of plank and blocks of wood

GENERAL BEHAVIOUR OF RESEARCH ASSISTANTS

- During Welcome and Stages 1-3
    - Behaviours of the research assistant can have a strong influence on participant behaviours and can alter the outcomes of the study (e.g., positive, open, warm behaviours may encourage speaking up, and vice versa). Unless these behaviours form part of the study manipulations (e.g., leaders that display openness or closedness to participants' views), it is thus important to consider the following:
        - Be *neutral* and *factual* towards the participant (think 'be professional'), neither too friendly nor too distant.
    - Prompts can be made when participants do not follow through on instructions (e.g., making an idea).
    - If experimental manipulations put requirements on behaviour, act in line with conditions throughout the welcome and stages 1-3.
- During the Debrief
    - The debrief has two purposes: clarify the true study intention and make the participant feel well. Hence, during the debrief: always be friendly, warm, open to questions, etc.

LABORATORY ENVIRONMENT

- A reception area for welcoming participants and paying out rewards
- Observation room with video recording facilities, or see-through mirror
- Quite experiment room(s)
- (optional) Provision of participant recruitment

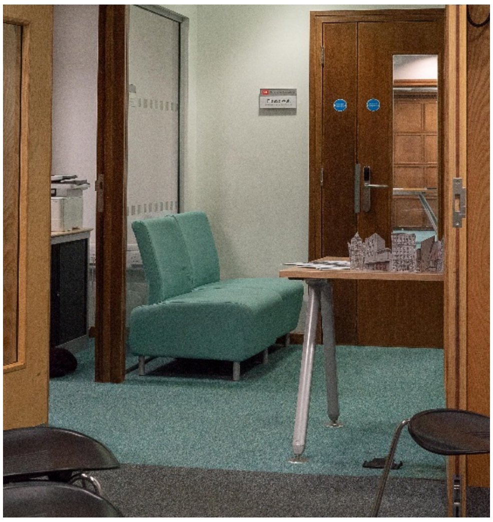

Example of a reception area (entrance).

MATERIALS

- 3 chairs
- A plank of wood (plywood, L: 120cm, W: 20cm, H:1.8cm)
- Blocks of wood (plywood, L: 3cm, W: 20cm, H:1.8cm)
- Table for the participant to sit at
- Questionnaire delivery method (i.e., iPad/PC, pen-and-paper).
- Duct tape
- Pens

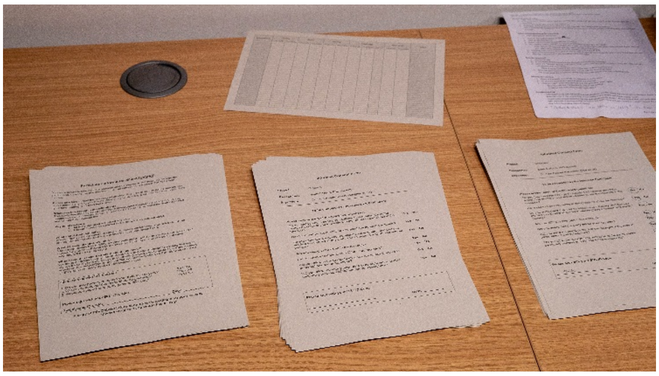

Study materials

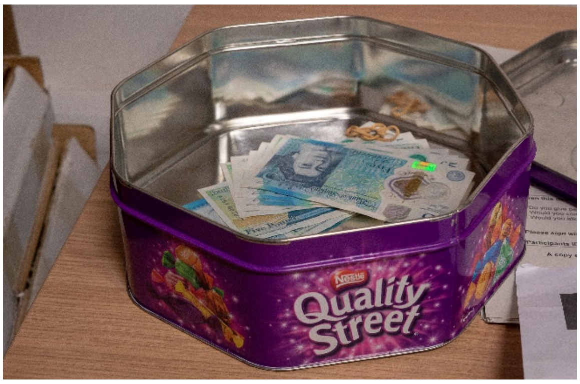

Participant reward

PREPARATION

RECEPTION AREA

1) Provide seating for participants arriving early
2) Ensure that the observation equipment cannot be seen from the reception area

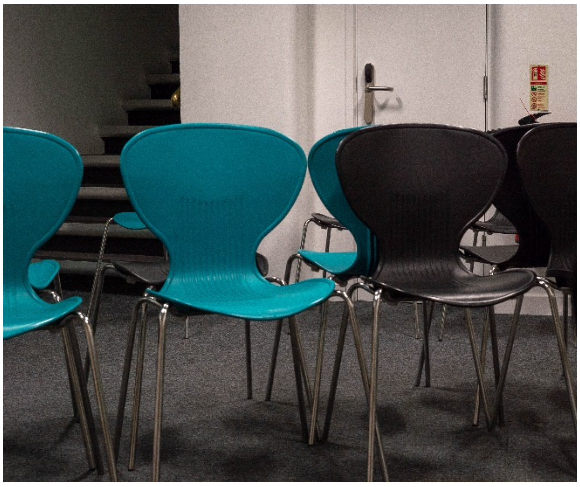

Reception area (seating)

OBSERVATION ROOM

1) Start the video recording of the Experiment room(s) used.
2) Ensure that the following are placed at a logical location, so you are ready to pick these up when you need them:
    1. A few working pens.
    2. Participant sign-up sheet (if laboratory provides advanced recruitment)
    3. Study information (BRIEF) / Informed consent sheet
    4. Study information (DEBRIEF)
    5. The Creativity-Feasibility form
    6. Payment confirmation sheet (if laboratory requires receipts)
    7. Money (i.e., participant reward)

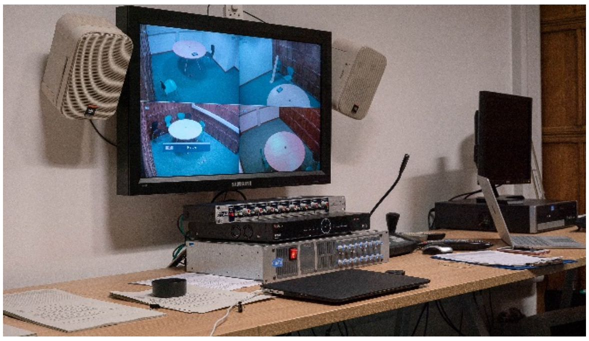

Observation Room equipment

EXPERIMENT ROOM

1) Set up:
    1. **Chairs**: Duct tape/mark two chairs to the ground at a distance on which the plank can be placed stably as a 'footbridge' between them.
    2. **Plank of woods**: place the plank casually standing upright against one of the chairs.
    3. **Blocks of wood**: place these casually at the base of the plank.
    4. **Table & chair**: Place a single chair at the table for the participant to sit at while writing (no duct taping). The participant needs to face the plank and chairs.
    5. **iPad**: ensure a link has been added to favourites, so it can be easily re-started for the next participant.

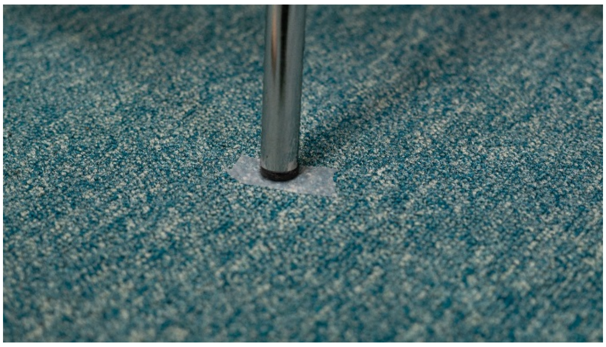

Location of chairs discretely marked to the ground

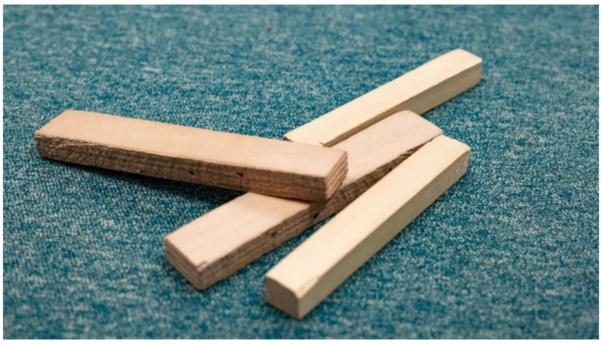

Blocks of wood

CHECKLIST

Requirements		OK?
**Laboratory environment**	A reception area for welcoming participants and paying out rewards	
	Observation room with video recording facilities, or see-through mirror	
	Quite experiment room(s)	
	Participant recruitment	
**Key decisions**	Manipulations: Which experimental manipulations are implemented and require tailoring of the protocol?	
	General behaviour of research assistants: how should research assistants behave in light of the experimental manipulations? (default = ‘professional’)	
**Logistics**	Ethical approval obtained	
	Laboratory space and time booked	
	Participant recruitment arranged	
	Research assistants allocated to timeslots	
**Materials**	A plank of wood (plywood, L: 120cm, W: 20cm, H:1.8cm)	
	Blocks of wood (plywood, L: 3cm, W: 20cm, H:1.8cm)	
	3 chairs	
	Table for the participant to sit at	
	Questionnaire delivery method (i.e., electronic, pen-and-paper).	
	Tape	
	Pens	
	Tracking sheet<br>(made in Excel to track: participant IDs, timeslots, safety voice behaviours, condition allocation, notes)	
	Participant reward (e.g., money)	
**Printing jobs**<br><br>(can be provided electronically)	Informed consent / brief form (printed in sufficient numbers)	
	Debrief form (printed in sufficient numbers)	
	Feasibility-creativity form for observing voice (printed in sufficient numbers)	
	Manual	
	Payment confirmation sheet (may come as laboratory facility)	
	Participant sign-up sheet (may come as laboratory facility)	

PROTOCOL STEPS

STAGE 1: PARTICIPANT WELCOME

Dependent on the laboratory facilities, participants may enter the laboratory in varying ways. However, a reception area with seating is recommended.

1) Participant arrives in reception area
2) When the participant time slot is about to start:
    1. Check whether the participant has arrived.
       (late participants should be denied participation, unless this can be accommodated)
    2. Request participant ID code (if the laboratory utilises this) and note down attendance on the participant sheet.
3) Register the participant and acquire informed consent
    1. Provide the 'Study Information (BRIEF) / Informed consent' sheet (see appendix A).
    2. Say:

"Here is some high-level information about what to expect in the study. Please read this carefully and answer the questions on the back of the sheet".

---

    3. Ensure the participant has agreed to all informed consent questions. (Participants are not allowed to participate without full agreement!).

Stage 2: 'Creativity' Task

1) Take the participant through to the experiment room.
2) Say:

"Please follow me."

---

3) Make the participant sit down.
4) Say:

"I am the research assistant for this study about creativity. The study involves 3 stages. First, you conduct a creativity task to develop creative ideas, then the feasibility and creativity of these ideas will be tested. Finally, there is a closing questionnaire."

---

5) Provide the Creativity Task and step back.
    1. This can be either paper-based, or via an online questionnaire tool (e.g., Qualtrics, SurveyMonkey, etc.)
    2. *To dodge questions, say:*

"The instructions make everything clear, please follow the instructions as provided."

---

6) Walk out of the experiment room for 5 minutes while the participant completes the task.
    1. Keep track of the time
        i) For iPad-based creativity tasks: these can be built to automatically transition (include a message to collect the research assistant) but make sure to keep track of participants as they may not collect the researcher.
        ii) For paper-and-pen-based creativity tasks: keep track of time yourself.

STAGE 3: DEMONSTRATION AND EVALUATION OF CREATIVE IDEAS

1) Take the 'Feasibility-Creativity form' with you.
2) Walk back into the Experiment room.

3) Explain the demonstration and evaluation of the creative ideas:
   1. Say:

"Okay. The next stage involves testing these ideas for two things: feasibility, either a yes or no, and creativity on a scale of 1-5 with 5 being high. However, your ideas will be tested by the next participant, and the ideas of the previous participant are tested."

---

4) Go through the list of creative ideas from the 'previous participant'. For each idea:
   1. Build: Let the participant build the idea.
   2. Engage: Engage with the idea briefly (as if quickly evaluating what is built by the participant).
   3. Feasibility: Ask whether the participant considers the idea feasible (request a: yes/no).
   4. Creativity: Ask whether the participant considers the idea creative (request a: 1-5).

5) Upon encountering the '*footbridge*' idea.
   1. Say:

"Hmm. That is actually pretty obvious, but I have not seen it before: could you please build a footbridge?"

---

   2. Follow the same order: build, engage, feasibility, creativity.
      i) If required, prompt participants to build a footbridge using the two chairs (unless they voice).
   3. Before walking towards the plank, say:

"I will now to test whether this is a footbridge."

---

6) The research assistant walks up to the plank across the two chairs and walks over it (unless this is an experimental condition!).

7) Observe voice vs silence:
   1. Note down on the form whether the participant speaks up about the plank being unsafe to walk on (before you have walked on it). For example, speaking up can sound like: "The plank stated a maximum weight", "That should be fine for a child (not you)"
   2. Voice should only be coded if it occurs between two time-points:
      i) After: the participant is asked to build the footbridge
      ii) Before: the RA is finished walking the plank and has stepped onto the ground.

Stage 4: Wrap-up Questionnaire

1) Finish demonstrating the creative ideas.
2) Say:

"For the final stage of the study, please complete this questionnaire."

---

3) Provide the wrap-up questionnaire
   1. If paper-and-pen-base: provide questionnaire and pen.
   2. If electronic: provide iPad/PC.
      Note: the electronic questionnaire can be built to automatically advanced from the Creativity Task during Stage 3, but ensure this takes sufficient time for the RA to not see the questionnaire prematurely.
4) Leave the room while the participant completes the questionnaire.

STAGE 5: DEBRIEF

Note: The intention of the debrief is to provide participants with a sense that they contributed to research, and explain to them that it was not about creativity, but speaking up about safety. This tends to work best when research assistants treat the debrief as an informal, open, and friendly conversation.

1) Take with you the Information sheet (DEBRIEF) and catch the participant before they come back to the waiting room and take them back to the experiment room.
2) Say:

"Thank you. This is the end of the study.
However, I'd like to just take 2 minutes to debrief you on the study.
I have to admit something: you may have guessed this study is actually not about creativity, but about how people speak up and the factors that influence this."

---

3) Provide Participant Debrief Information Sheet. Say:

"Please take your time to read this and ask me any question you would have."

---

4) Discuss questions with the participants (or refer to the lead researcher).
5) Ensure the participant completes the debrief questions.
   1. If a participant considers the research unethical: talk this through with them (they often misread the questions) and inform the lead researcher.
6) Take participants back to the reception area. Say:

Okay. Unless you have any remaining questions, let's go back and get you the reward for your time. Please follow me.

---

7) Pay the participant their reward.

REFERENCES

Bienefeld, N., & Grote, G. (2012). Silence that may kill: When aircrew members don't speak up and why. *Aviation Psychology and Applied Human Factors*, *2*(1), 1–10. https://doi.org/10.1027/2192-0923/a000021

Moorhead, G., Ference, R., & Neck, C. P. (1991). Group Decision Fiascoes Continue: Space Shuttle Challenger and a Revised Groupthink Framework. *Human Relations*, *44*(6), 539–550. https://doi.org/10.1177/001872679104400601

Reader, T., & O'Connor, P. (2014). The Deepwater Horizon explosion: Non-technical skills, safety culture, and system complexity. *Journal of Risk Research*, *17*, 405–424.

Tarnow, E. (1999). Self-destructive obedience in the airplane cockpit and the concept of obedience optimization. In *Obedience to authority* (pp. 125–138). Psychology Press.

Appendix A: Brief form and Informed consent (paper version)

Participant Information Sheet (Brief)

You are being invited to take part in a research study. Before deciding to participate it is important for you to understand why the research is being done and what it will involve. Please take time to read the following information about the study and don't feel rushed.

What is this research about?
You will engage in a task about creativity and associated factors.

Who is doing this research?
This study is led by [name], [role] at [department], [institution].

Why have you asked me to participate?
You have been recruited via [recruitment method].

What will participation involve?

- Participation involves a number of tasks around creativity
- Some participant may receive a closing questionnaire or interview
- A video recording may be made

How long will participation take?
Up to 30 minutes.

What about confidentiality?
Your data will be saved anonymously. Video recordings will be analysed by the experimenter and not be shared beyond the research team (unless you give explicit and voluntary approval after the study).

If you are willing to participate,
then please sign the Consent Form on the reverse of this page.

You can keep a copy of this Information Sheet for your records
(please enquire)

Informed Consent Form

Project: Study on Creativity

Researcher: [names]

Supervisor: [names]

To be completed by the Research Participant

Please answer each of the following questions:

Do you feel you have been given sufficient information about the research to enable you to decide whether or not to participate in the research?	**Yes**	**No**
Have you had an opportunity to ask questions about the research?	**Yes**	**No**
Do you understand that your participation is voluntary, and that you are free to withdraw at any time, without giving a reason, and without penalty?	**Yes**	**No**
Are you willing to take part in the research?	**Yes**	**No**
Are you aware that the study will be video recorded?	**Yes**	**No**
Will you allow the research team to use anonymized quotes in presentations and publications?	**Yes**	**No**
Will you allow the anonymized data to be archived, to enable secondary analysis and training future researchers?	**Yes**	**No**

The lab employs a no-name policy.

Please sign with your *ID Code*:

______________________________ **Date:** __________

APPENDIX B: DEBRIEF FORM (PAPER VERSION)

Participant Information Sheet (Debrief)

You were invited to take part in a research study. However, to not invalidate findings (by participants knowing the true intention), the true nature of this research had to be kept hidden.

Please take time to read the following information. Feel free to discuss issues with anyone, and if there is anything which is not clear or if you have any questions, feel free to ask. Take your time reading, and don't feel rushed.

What was this research *actually* about?
This study was not only about creativity. It investigated whether people speak up about unsafe events. To promote safety, it investigated 'speaking up' about the 'footbridge' idea and factors that influence this.

Key to understand:

1. All materials used are tested and safe.
2. Research assistants played a role following an outlined script.

What about confidentiality?
This remains unchanged. We will treat your data video recording in strictest confidentiality. Your video will not be shared beyond the research team unless you give permission below.

What if I have changed my mind?
You are completely free to withdraw your participation without any consequence, or loss of reward for your time. Please indicate this to us if this case. Your answers and video recording will then be destroyed.

What if I want to raise a concern about this study?
We are open to listen to any concerns you have and are committed to working with you to resolve these (after all, this is what we study). If you want to raise any remaining issues, please contact the experimenter's supervisor ([email]), the [name laboratory] ([email]) or the [name institution] ethics committee ([email]).

Given this debrief information:

1. Do you give permission to use your data for analyses?	Yes / No
2. Would you consider this research to be *un*ethical?	Yes / No
3. Would you allow future participants to take part?	Yes / No

The lab employs a no-name policy.
Please sign with your ID code:

Participants ID code: ______________________ **Date: __________**

A copy of this Debrief Information Sheet is available for your records
(please enquire if you would like a copy)

APPENDIX C: THE CREATIVITY-FEASIBILITY FORM (PAPER VERSION)

Participant ID	Shelfing		Mirror		Juggling		Footbridge			Piece of art		Notes
	Creativity	Feasibility	Creativity	Feasibility	Creativity	Feasibility	Creativity	Feasibility	Voice?	Creativity	Feasibility	

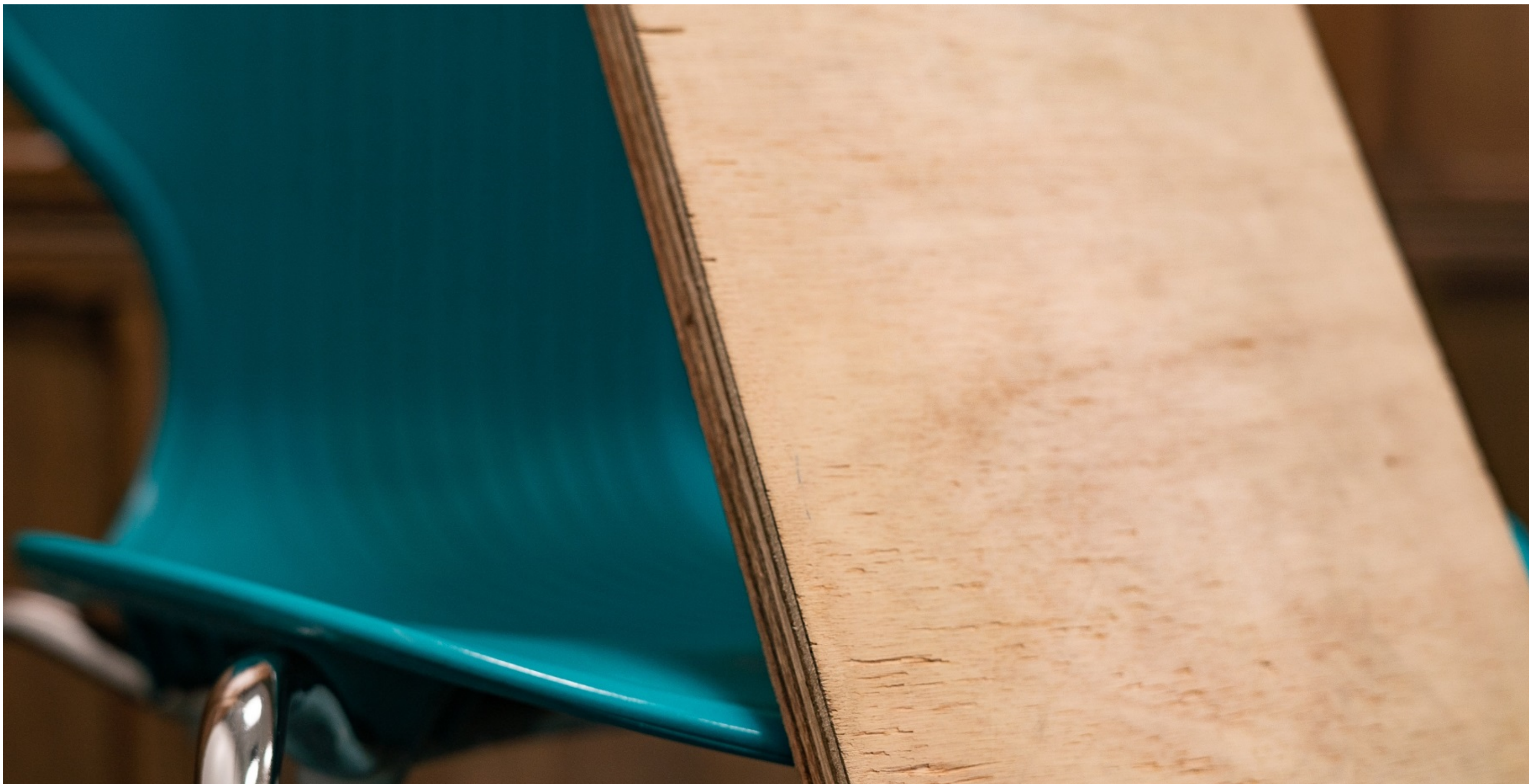

© Mark C Noort, 2019
